# Supplementary material for: Addressing the need for interactive, efficient, and reproducible data processing in ecology with the datacleanr R package
Source: PLoS One. 2022 May 12;17(5):e0268426. doi: 10.1371/journal.pone.0268426 (PMC9098071; doi:10.1371/journal.pone.0268426)
Supplement: S1 File — An overview of the package’s functionalities with animated examples of every feature. (HTML) [file pone.0268426.s001.html]

Addressing the need for interactive, efficient and reproducible data processing in ecology with the datacleanr R package


# Addressing the need for interactive, efficient and reproducible data processing in ecology with the datacleanr `R` package

#### Alexander Hurley, Richard L. Peters, Christoforos Pappas, David Steger, Ingo Heinrich

#### 3/15/2022

# datacleanr

`datacleanr` is a flexible and efficient tool for **interactive** data cleaning, and is inherently interoperable, as it seamlessly integrates into **reproducible** data analyses pipelines in `R`.

It can deal with nested **tabular**, as well as **spatial** and **time series** data.

## Installation

The latest release on CRAN can be installed using:

```
install.packages("datacleanr")
```

You can install the development version of `datacleanr` with:

```
remotes::install_github("the-hull/datacleanr")
```

**If you are using macOS, please make sure you have `XQuartz` installed, especially if you’ve recently updated your system.** **See these instructions here: https://CRAN.R-project.org/bin/macosx/**

## Design

`datacleanr` is developed using the shiny package, and relies on informative summaries, visual cues and interactive data selection and annotation. All data-altering operations are documented, and converted to valid `R` code (**reproducible recipe**), that can be copied, sent to an active `RStudio` script, or saved to disk.

There are **four tabs** in the app for these tasks:

- **Set-up & Overview**: define nesting structure based on (multiple) groups.
- **Filtering**: use `R` expression to filter/subset data.
- **Visual Cleaning and Annotating**: generate bivarirate (time series) plots and maps, as well as highlight and annotate individual observations. Cycle through nested groups to expedite exploration and cleaning. Histograms of original vs. ‘cleaned’ data can be generated.
- **Extract**: generate reproducible recipe and define outputs. **`dcr_app` also returns all intermediate and final outputs invisibly to the active `R` session for later use (e.g. when batch processing)**

Note, maps require columns `lon` and `lat` (X and Y) in decimal degrees in the data set to render.

## Additional features

- **Grouping**: the grouping defined in the “Set-up and Overview” tab is carried forward through the app. These groups can be used to cycle through nested/granular data, and considerably speed up exploration and cleaning. These groups are also available for filtering (Filtering tab), where filter expressions can be scoped to group level (i.e. no groups, individual, all groups).
- **Interoperability**: when a logical (`TRUE`\`FALSE`) column named `.dcrflag` is present, corresponding observations are rendered with different symbols in plots and maps. Use this feature to validate or cross-check external quality control or outlier flagging methods.
- **Batching**: If data sets are too large, or too deeply nested (e.g. individual, plot, site, region, etc.), we recommend a split-combine approach to expedite the processing.

```
# prepare data into species sub-sets
iris_split <- split(x = iris,
                    f = iris$Species)
# run for each species
dcr_iris <- lapply(iris_split, 
                   function(split){
                       datacleanr::dcr_app(split)
                   })
```

## Getting started

The documentation for (`?dcr_app()`) explains the basic use and all features. Throughout the app, there are conveniently-placed help links that provide details on features.

## Demonstration

Launch `datacleanr`’s interactive app with `dcr_app()`. The following examples demonstrate basic use and highlight features across the four app tabs.

### 1. Set-up & Overview

Define the grouping structure (used throughout app for scoping filters and plotting), and generate an informative overview.

```
library(datacleanr)

# group by species
dcr_app(iris)
```

### 2. Filtering

Add/Remove filter statement boxes, and apply (valid) expressions - either to the entire data set, or scoped to individual groups. Filtering relies on `R` expressions passed to `dplyr::filter()`, so, for example, valid statements for `iris` are:

```
    Species == 'setosa'
    Species %in% c('setosa','versicolor')
    Sepal.Width > quantile(Sepal.Width, 0.05)
```

Any function returning a logical vector (i.e. `TRUE`/`FALSE`), can be employed here!

### 3. Visualizing and annotating

Interactive visualization allow seamless scrolling, panning and zooming to select and annotate individual observations (or sections with lasso/box select tool). Show and hide groups using the group selection table (left) or the legend (right).

#### 3.1 General highlighting and annotating

#### 3.2 Using `.dcrflag` to interface with external QA/QC

```
library(datacleanr)
library(dplyr)

iris_mod <- iris %>%
group_by(Species) %>%
  # .dcrflag provides additional visual cue in visualization tab
  # based on TRUE/FALSE 
mutate(.dcrflag = Sepal.Width < quantile(Sepal.Width, 0.05))


dcr_app(iris_mod)
```

#### 3.3 Time Series

Any `numeric` or `POSIXct` column (in X or Y dimension) can be used to visualize time series. Use the `Toggle Lines` button above the plot to facilitate exploration.

**Example 1**:

```
library(dplyr)

dplyr::glimpse(treering)
tree_df <- data.frame(year = -6000:1979,
           val = treering)

# make synthetic data
tree_data <- list(tree_A = tree_df,
                  tree_B = tree_df %>% 
                      mutate(val = val + rnorm(nrow(.), 0.5, 0.2)),
                  tree_C = tree_df %>% 
                      mutate(val = val + rnorm(nrow(.), mean = -0.03, 0.1))) %>% 
    bind_rows(.id = "tree")

# group by tree and inspect
dcr_app(tree_data)
```

> (Note, selections are arbitrary and for demonstration only)

**Example 2**:

> No GIF - but similar execution as above

```
library(dplyr)
library(lubridate)
data("storms", package = "dplyr")

storms_mod <- storms %>% 
    mutate(timestamp = lubridate::ymd_h(paste(year, month, day, hour)))

# Group by name (198 groups)
# Check "Emily"
dcr_app(storms_mod)
```

#### 3.4 Spatial

Interactive maps rely on Mapbox for plotting. Therefore, you will need to make an account, from which an access token needs to be copied into your `.Renviron` (e.g. `MAPBOX_TOKEN=your_copied_token`). A simple way to do this is using the convenient `usethis` package to access the file:

```
usethis::edit_r_environ()
```

Select columns `lon` and `lat` for plotting to get started.

**Example 1**

```
library(datacleanr)
library(dplyr)

airport_data <- read.csv('https://plotly-r.com/data-raw/airport_locations.csv') %>%
    rename(lon = long)

# group by state
dcr_app(airport_data)
```

> No GIF - but see this animtion on the Github repository

**Example 2**

> No GIF - but identical execution as above

```
library(dplyr)
library(lubridate)
data("storms", package = "dplyr")


storms_mod <- storms %>% 
    rename(lon = long)

# Group by name (198 groups)
# Check "Bonnie"
dcr_app(storms_mod)
```

### 4. Extract (Reproducible Recipe)

All grouping, filtering and selections/annotations are translated to `R` code, which can be sent to an `RStudio` script, copied to the clipboard, or - when `dcr_app` is launched with a file path - save options are made available. For large selections/annotations we recommend saving the script separately, and sourcing it (i.e. `source("your_datacleanr_script.R")`) during later analyses.

**Caution: When selections / annotations are greater than ~ 1000 points, it is recommended to use `datacleanr` with an `*.RDS` file (see below). This is because the resulting Reproducible Recipe (script) can slow down the RStudio IDE, if it has more than a few thousand lines.The next version of `datacleanr` will allow choosing between script-only recipes, and the option with an the intermediate file for storing annotations. Both approaches with their current implementation are shown shown below.**

**Example 1**

Launching with an object from `R`:

```
library(datacleanr)
dcr_app(iris)
```

And output from extract tab:

```
# datacleaning with datacleanr (0.0.1)
# ##------ Wed Oct 07 12:54:03 2020 ------##

library(dplyr)
library(datacleanr)

#  adding column for unique IDs;
iris$.dcrkey <- seq_len(nrow(iris))


iris <- dplyr::group_by(iris, Species)

#  stats and scoping level for filtering
filter_conditions <- structure(list(filter = "Sepal.Width > 2.7", grouping = list(NULL)), row.names = c(NA, 
    -1L), class = c("tbl_df", "tbl", "data.frame"))

#  applying (scoped) filtering by groups;
iris <- datacleanr::filter_scoped_df(dframe = iris, condition_df = filter_conditions)

#  observations from manual selection (Viz tab);
iris_outlier_selection <- structure(list(.dcrkey = c(15L, 16L, 19L, 34L), .annotation = c("", "", "", 
    "")), class = "data.frame", row.names = c(NA, -4L))

#  create data set with annotation column (non-outliers are NA);
iris <- dplyr::left_join(iris, iris_outlier_selection, by = ".dcrkey")

# remove comment below to drop manually selected obs in data set;
# iris  <- iris %>% dplyr::filter(is.na(.annotation))
```

**Example 2**

Launching with an `.RDS` from disk:

```
saveRDS(iris, file = "./testiris.Rds")

library(datacleanr)
dcr_app("./testiris.Rds")
```

---
