# Supplementary material for: Addressing the need for interactive, efficient, and reproducible data processing in ecology with the datacleanr R package
Source: PLoS One. 2022 May 12;17(5):e0268426. doi: 10.1371/journal.pone.0268426 (PMC9098071; doi:10.1371/journal.pone.0268426)

PLOS ONE

Addressing the need for interactive, efficient and reproducible data processing in ecology with the datacleanr R application

--Manuscript Draft--

**Manuscript Number:**	PONE-D-21-05607
**Article Type:**	Research Article
**Full Title:**	Addressing the need for interactive, efficient and reproducible data processing in ecology with the datacleanr R application
**Short Title:**	Interactive and reproducible data processing with datacleanr
**Corresponding Author:**	Alexander Hurley, Ph.D.<br>Helmholtz-Zentrum Potsdam GFZ: Deutsches Geoforschungszentrum Potsdam<br>Potsdam, Brandenburg GERMANY
**Keywords:**	reproducible analyses; outlier assessment; quality control; time series processing
**Abstract:**	Ecological research, just as all Earth System Sciences, is becoming increasingly data-rich. Tools for processing of “big data” are continuously developed to meet corresponding technical and logistical challenges. However, even at smaller scales, data sets may be challenging for individual researchers when best practices in data exploration, quality control and reproducibility are to be met. This can occur when conventional methods, such as generating and assessing diagnostic visualizations or tables, become unfeasible due to time and practicality constraints. Interactive processing can alleviate this issue, and is increasingly utilized to ensure that large data sets are diligently handled. However, recent interactive tools rarely enable data manipulation, may not generate reproducible outputs, or are typically data/domain-specific, and potentially limited to a (commercial) platform. We developed datacleanr , an interactive tool that facilitates best practices in data exploration, quality control (e.g., outlier assessment) and flexible processing for multiple tabular data types, including time series and georeferenced data. The application is open-source, and based on the R programming language. A key functionality of datacleanr is the “reproducible recipe” - a translation of all interactive actions into R code, which can be integrated into existing analyses pipelines. This enables researchers experienced with script-based workflows to utilize the strengths of interactive processing without sacrificing their usual work style or functionalities from other (R) packages. We demonstrate the application’s utility by addressing two common issues during ecological data analyses, namely 1) identifying problematic structures and artefacts in hierarchically nested data, and 2) preventing excessive loss of data from ‘coarse,’ code-based filtering of time series. Ultimately, with datacleanr we aim to improve researchers’ workflows and increase confidence in and reproducibility of their results.
**Order of Authors:**	Alexander Hurley, Ph.D.
	Richard L Peters
	Christoforos Pappas
	David Steger
	Ingo Heinrich
**Additional Information:**	
**Question**	**Response**
**Financial Disclosure**<br><br>Enter a financial disclosure statement that describes the sources of funding for the work included in this submission. Review the submission guidelines for detailed requirements. View published research	AGH and IH were supported through the Helmholtz-Climate-Initiative (HI-CAM), funded by the Helmholtz Initiative and Networking Fund (https://www.helmholtz.de/en/about-us/the-association/initiating-and-networking/); the authors are responsible for the content of this publication. RLP acknowledges support of the Swiss National Science Foundation (http://www.snf.ch/), Grant P2BSP3_184475. The funders had no role in study design, data collection and analysis, decision to publish, or preparation of the manuscript.

Powered by Editorial Manager® and ProduXion Manager® from Aries Systems Corporation

articles from *PLOS ONE* for specific examples.<br><br>This statement is required for submission and **will appear in the published article** if the submission is accepted. Please make sure it is accurate.<br><br>**Unfunded studies**<br>Enter: *The author(s) received no specific funding for this work.*<br><br>**Funded studies**<br>Enter a statement with the following details:<br>• Initials of the authors who received each award<br>• Grant numbers awarded to each author<br>• The full name of each funder<br>• URL of each funder website<br>• Did the sponsors or funders play any role in the study design, data collection and analysis, decision to publish, or preparation of the manuscript?<br>• **NO** - Include this sentence at the end of your statement: *The funders had no role in study design, data collection and analysis, decision to publish, or preparation of the manuscript.*<br>• **YES** - Specify the role(s) played.<br><br>* typeset	
**Competing Interests**<br><br>Use the instructions below to enter a competing interest statement for this submission. On behalf of all authors, disclose any competing interests that could be perceived to bias this work—acknowledging all financial support and any other relevant financial or non-financial competing interests.<br><br>This statement is required for submission and **will appear in the published article** if the submission is accepted. Please make sure it is accurate and that any funding sources listed in your Funding Information later in the submission form are also declared in your Financial Disclosure statement.	The authors have declared that no competing interests exist.

View published research articles from *PLOS ONE* for specific examples.<br><br>**NO authors have competing interests**<br><br>Enter: *The authors have declared that no competing interests exist.*<br><br>**Authors with competing interests**<br><br>Enter competing interest details beginning with this statement:<br><br>*I have read the journal's policy and the authors of this manuscript have the following competing interests: [insert competing interests here]*<br><br>* typeset	
**Ethics Statement**<br><br>Enter an ethics statement for this submission. This statement is required if the study involved:<br><br>• Human participants<br>• Human specimens or tissue<br>• Vertebrate animals or cephalopods<br>• Vertebrate embryos or tissues<br>• Field research<br><br>Write "N/A" if the submission does not require an ethics statement.<br><br>General guidance is provided below. Consult the submission guidelines for detailed instructions. **Make sure that all information entered here is included in the Methods section of the manuscript.**	N/A

**Format for specific study types**<br><br>**Human Subject Research (involving human participants and/or tissue)**<br>• Give the name of the institutional review board or ethics committee that approved the study<br>• Include the approval number and/or a statement indicating approval of this research<br>• Indicate the form of consent obtained (written/oral) or the reason that consent was not obtained (e.g. the data were analyzed anonymously)<br><br>**Animal Research (involving vertebrate animals, embryos or tissues)**<br>• Provide the name of the Institutional Animal Care and Use Committee (IACUC) or other relevant ethics board that reviewed the study protocol, and indicate whether they approved this research or granted a formal waiver of ethical approval<br>• Include an approval number if one was obtained<br>• If the study involved *non-human primates*, add *additional details* about animal welfare and steps taken to ameliorate suffering<br>• If anesthesia, euthanasia, or any kind of animal sacrifice is part of the study, include briefly which substances and/or methods were applied<br><br>**Field Research**<br><br>Include the following details if this study involves the collection of plant, animal, or other materials from a natural setting:<br>• Field permit number<br>• Name of the institution or relevant body that granted permission	
**Data Availability**<br><br>Authors are required to make all data underlying the findings described fully available, without restriction, and from the time of publication. PLOS allows rare exceptions to address legal and ethical concerns. See the PLOS Data Policy and FAQ for detailed information.	Yes - all data are fully available without restriction

Powered by Editorial Manager® and ProduXion Manager® from Aries Systems Corporation

A Data Availability Statement describing where the data can be found is required at submission. Your answers to this question constitute the Data Availability Statement and **will be published in the article**, if accepted.<br><br>**Important:** Stating 'data available on request from the author' is not sufficient. If your data are only available upon request, select 'No' for the first question and explain your exceptional situation in the text box.<br><br>Do the authors confirm that all data underlying the findings described in their manuscript are fully available without restriction?	
**Describe where the data may be found in full sentences. If you are copying our sample text, replace any instances of XXX with the appropriate details.**<br><br>• If the data are **held or will be held in a public repository**, include URLs, accession numbers or DOIs. If this information will only be available after acceptance, indicate this by ticking the box below. For example: *All XXX files are available from the XXX database (accession number(s) XXX, XXX.).*<br>• If the data are all contained **within the manuscript and/or Supporting Information files**, enter the following: *All relevant data are within the manuscript and its Supporting Information files.*<br>• If neither of these applies but you are able to provide **details of access elsewhere**, with or without limitations, please do so. For example:<br><br>*Data cannot be shared publicly because of [XXX]. Data are available from the XXX Institutional Data Access / Ethics Committee (contact via XXX) for researchers who meet the criteria for access to confidential data.*<br><br>*The data underlying the results presented in the study are available from (include the name of the third party*	All data necessary for replication are publicly available. The presented software is available from an online repository https://github.com/the-Hull/datacleanr) and via CRAN (https://cran.r-project.org/package=datacleanr). Eddy covariance data are available online from the FLUXNET2015 webpage (http://fluxnet.fluxdata.org/data/fluxnet2015-dataset/). The Berlin street and park tree data is available at https://daten.berlin.de/. The data to reproduce the profiling and time series cleaning example are available at Zenodo via https://doi.org/10.5281/zenodo.4550727

and contact information or URL).<br>• This text is appropriate if the data are owned by a third party and authors do not have permission to share the data.<br><br>* typeset	
Additional data availability information:	

Manuscript

Click here to access/download;Manuscript;PLOS_datacleanr_manuscript_Feb

Addressing the need for interactive, efficient and reproducible data processing in ecology with the `datacleanr` R application

Alexander G. Hurley[1,✉], Richard L. Peters[2], Christoforos Pappas[3,4], David Steger[1,5], and Ingo Heinrich[1]

19 February, 2021

[1] GFZ German Research Centre for Geosciences, Section ‘Climate Dynamics and Landscape Evolution,’ Potsdam, Germany

[2] Laboratory of Plant Ecology, Department of Plants and Crops, Faculty of Bioscience Engineering, Ghent University, Coupure links 653, B-9000 Ghent, University of Ghent, Belgiums

[3] Centre d’étude de la forêt, Université du Québec à Montréal, C.P. 8888, Succursale Centre-ville, Montréal, QC, H3C 3P8, Canada

[4] Département Science et Technologie, Téluq, Université du Québec, 5800 rue Saint-Denis, Bureau 1105, Montréal, QC, H2S 3L5, Canada

[5] Humboldt-Universität zu Berlin, Berlin

✉Correspondence: Alexander G. Hurley <hurley@gfz-potsdam.de>

Short title: Interactive and reproducible data processing with `datacleanr`

Target Journal: PLOS One

Abstract

Ecological research, just as all Earth System Sciences, is becoming increasingly data-rich. Tools for processing of "big data" are continuously developed to meet corresponding technical and logistical challenges. However, even at smaller scales, data sets may be challenging ~~for individual researchers~~ when best practices in data exploration, quality control and reproducibility are to be met. This can occur when conventional methods, such as generating and assessing diagnostic visualizations or tables, become unfeasible due to time and practicality constraints. Interactive processing can alleviate this issue, and is increasingly utilized to ensure that large data sets are diligently handled. However, recent interactive tools rarely enable data manipulation, may not generate reproducible outputs, or are typically data/domain-specific, and potentially limited to a (commercial) platform. We developed `datacleanr`, an interactive tool that facilitates best practices in data exploration, quality control (e.g., outlier assessment) and flexible processing for multiple tabular data types, including time series and georeferenced data. The application is open-source, and based on the `R` programming language. A key functionality of `datacleanr` is the "reproducible recipe" - a translation of all interactive actions into `R` code, which can be integrated into existing analyses pipelines. This enables researchers experienced with script-based workflows to utilize the strengths of interactive processing without sacrificing their usual work style or functionalities from other (`R`) packages. We demonstrate the application's utility by addressing two common issues during ecological data analyses, namely 1) identifying problematic structures and artefacts in hierarchically nested data, and 2) preventing excessive loss of data from 'coarse,' code-based filtering of time series.

Ultimately, with `datacleanr` we aim to improve researchers' workflows and increase confidence in and reproducibility of their results.

Introduction

Ecology, just as all Earth system sciences, is increasingly data-rich [e.g., 1]. These data are a boon for novel inferences, and increasingly inform decision making [2,3], for example, through databases from coordinated efforts that facilitate synoptic studies of carbon fluxes [e.g., FLUXNET, 4] and stocks [5] or ecosystem functioning [e.g., via trait databases like TRY, 6]. Low-cost monitoring and sensing solutions have also immensely increased the amount of data individual researchers can produce [e.g., 7]. However, the data deluge - often from heterogeneous sources - introduces new logistical and computational challenges for researchers [7,8] wanting to maintain best practices in data analyses, reproducibility and transparency [see frameworks in 9,10]. It is clear that we need not only frameworks, but also flexible tools to deal with the ever-increasing, heterogeneous data and corresponding issues.

Paramount to any analyses is ensuring the validity of input data through adequate exploration and quality control, which allows identifying any idiosyncrasies, outliers or erroneous structures. However, with growing data volumes this becomes increasingly difficult. Indeed, several definitions establish "big data" at the threshold where single entities (i.e. researchers, institutions, disciplines) are no longer able to manage and process a given data set due to its size or complexity [e.g., 11,12]. Yet, several current research applications in ecology and Earth system science require handling more than Gigabyte-scale data and regularly lead to the development of dedicated and domain-specific processing pipelines and

tools, e.g., processing of raw data from FLUXNET [4] or automating data assimilation from long-term experiments [13].

Individual ecologists, however, frequently encounter data sets smaller than this, which nonetheless challenge the feasibility of common data processing and exploration methods. These include the best practice examples of generating static diagnostic/summary visualizations, statistics and tables for detecting problematic observations [e.g., 14,15]. Data sets of this scale are termed "high-volume," rather than "big," for purposes of this study. Issues with these data often arise when the dimensions and data types require numerous of the aforementioned items (e.g., visualizations), and their individual assessment becomes unfeasible due to time and practicality constraints, even when their generation can be largely automated. Hence, they can pose a challenge even for experienced researchers adept at script-based analyses, if convenient tools do not exist or are financially inaccessible. For instance, over-plotting may require generating several static visualizations for nested categorical levels, such as branch, individual tree and forest stand, or for spatial granularity, such as plot, site and region. Furthermore, time series from monitoring equipment may show issues related to sensor drift, step-shifts, or random sensor errors. While gap-filtering, trend-adjustment and outlier-removal algorithms exist for these circumstances [e.g., 16,17], subsequent manual checking is usually still advised, leading to similar issues as above. For time series, in particular, problematic periods (e.g., systematically-occurring sensor errors) may be removed entirely for convenience in code-based processing; by contrast, interactive engagement down to individual observations may allow applying more diligence and retaining more data.

Ideally, researchers should be able to engage with their data, across scales and dimensions as diligently as needed, with as little effort as possible. Accordingly, interactive processing is increasingly called for and deemed critical [18] for ensuring best practices in data exploration, and quality control when dealing with high-volume data and beyond [e.g., 19,20]. Indeed, interactive exploration is increasingly provided through open-source graphing frameworks (e.g., plotly, highcharts) and/or commercially-licensed software (e.g., Excel, Tableau). However, actual data manipulation, and especially the generation of subsequent outputs that are fully reproducible, are far less common features; this could potentially stimulate reluctance for sharing analysis code [e.g., 21]. Further issues can arise when outputs are platform/software dependent and thus not easily incorporated with other widely-used languages, such as `R` [22] or `python` [23]. Reproducible and interactive processing is, therefore, typically linked to method-specific workflows within research domains, for instance, to annotate images [e.g., 24], acoustic files [e.g., 25], or explore spatial and time series data [e.g., 20,26].

There is a clear need for interactive tools that can facilitate best practices in processing heterogeneous, high-volume data, while enabling interoperability with reproducible workflows. To address this, we developed `datacleanr`: an open-source `R`-based tool for rapid exploration, data filtering as well as interactive visualization and annotation of various data types, including spatial (georeferenced) and time series observations. `datacleanr` is designed to fit in existing, scripted processing (`R`) pipelines, without sacrificing the benefits of interactivity: this is achieved through features that allow validating the results of previous quality control, and by generating a code script to repeat any interactive operation - this can

be slotted into existing workflows, and `datacleanr`'s output can hence be directly used for subsequent reproducible analyses.

Below we provide an overview of the application. Additionally, we demonstrate `datacleanr`'s utility with two ecology-based use-cases addressing common issues during data processing: 1) Identifying problematic data structures and artefacts using an urban tree survey, where data is nested by species, street and city district. 2) Preventing excessive loss of data from "coarse," code-based filtering in messy time series of sap flow data, bolstering subsequent analyses.

Lastly, we provide an outlook for future developments and conclude by inviting the community to contribute to further increase `datacleanr`'s capabilities and reach.

`datacleanr` overview

Availability

Stable releases of `datacleanr` are available on the Comprehensive `R` Archive Network (CRAN; `install.packages("datacleanr")`) and Zenodo. Latest developments are on a dedicated repository (www.github.com/the-hull/datacleanr), where installation instructions for all sources and animated demonstrations with test data are provided. `datacleanr` is available under a GPL-3 license.

Capabilities

`datacleanr` is an interactive`R` package for processing high-volume data, and it caters to best practices in data exploration, processing and reproducibility. It uses the `shiny` [27] and `plotly` [28] packages to generate a web browser-based graphical user interface. It can

therefore display up to approximately 2 million observations, above which modern web-browsers begin reaching their limits. It has four modules represented in application tabs, which are documented using intuitively-placed help links and package documentation: 1) Set-up and Overview (grouping and exploration), 2) Filtering, 3) Visual Cleaning and Annotating, 4) Extraction (reproducible recipe). The application is launched with `datacleanr::dcr_app()` in `R` (run `?datacleanr::dcr_app()` for help).

The chart in Fig 1 shows the `datacleanr` workflow across the four modules (A-D) with optional pre- and post-processing with external algorithms. Users are encouraged to cycle through multiple grouping structures, filters and variable combinations to get adequately acquainted with their data. The functions of individual tabs are discussed in detail below.

Figure 1: ***Conceptual workflow for*** **`datacleanr`** ***across its four processing modules.*** *Nested data (e.g., by plot, site, region) can be assessed by setting the grouping structure in A) and targeted ("scoped") filters or visualizing individual groups accordingly. The schematic here illustrates the potential for including* `datacleanr` *into an existing workflow, for example, with prior determination of outliers using external algorithms (by appending a logical* `TRUE/FALSE` *column named* `.dcrflag`*), interactive exploration and processing, and informing subsequent analyses via interactively annotated data (*`.annotation` *column in output).*

Set-up and overview

`datacleanr` facilitates processing of nested data through defining a grouping structure to the level of interest (e.g., by selecting species, plot and region). The structure is available during targeted filtering (scoping; see *Filtering*) and visual cleaning (see Figs 6-8) after "Set and Start" is clicked. Once the grouping is set, a convenient dataset summary can be generated [via 29], highlighting duplicates, missingness, and distribution of each variable by clicking on "Generate Overview."

Filtering

Filtering is done by adding filter statement boxes. Statements can be applied to the entire data set, or targeted to specific groups using a "scoped" (i.e. group-specific) filter. The application's interactivity allows reviewing the impact of filters through text cues on the tab, and by iterating between settings and visualizations (across several variable combinations). This is more efficient than (re-)generating individual, static figures, and highlights which data will be excluded. The result of a quantile-based threshold filter implemented in `datacleanr`, as used e.g., in TRY [6] or BAAD [30], is illustrated in Fig 2.

Figure 2: ***Example application of statistical filtering.*** *A percentile threshold filter (0.01 and 0.99) is applied to the full range or scoped to groups (panels, see bold text) on the x-variable. Trait data from BAAD [30] is used to illustrate the impact on bivariate relationships across plant functional types, where the gray shading and text labels indicate the filtered variable space. Note, the figure was not generated in `datacleanr`.*

Any statement evaluating to `TRUE` or `FALSE` and using the dataset's column names can be used, as shown below. That is, let `example_numeric_variable` and `example_categorical_variable` be column names, then following statements are valid and can be supplied in a filter statement box:

```
# simple logical filter
example_numeric_variable > 3

# using expressions to define thresholds
## percentile/rank based
example_numeric_variable > quantile(example_numeric_variable, 0.01)
## dispersion based (median absolute distance)
example_numeric_variable > median(example_numeric_variable) -
  3 * mad(example_numeric_variable)

# example for subsetting
example_categorical_variable == "SpeciesA"
example_categorical_variable %in% c("SpeciesA", "SpeciesB")
```

Visual cleaning and annotating

Interactive visualizations [via 28] are based on bivariate scatter or time series plots with optional third dimension represented by point size. Spatial data can displayed on interactive map tiles, if columns named `lon` (longitude) and `lat` (latitude) are present in decimal degrees. Example visualizations for currently supported data types are in Fig 3.

Figure 3: ***Examples of interactive visualizations.*** *Panels show a subset of hourly time series of latent heat fluxes (A) from all Swiss FLUXNET2015 sites [4], spatial data illustrating sample locations for BAAD (B) and the relationship between stem diameter and height (C) with plant traits from BAAD [30]. Colors represent the grouping structure defined in the "Set-up" operation (A: Swiss sites; B, C: functional types). Map created with Mapbox and OpenStreetMap and their data sources. Satellite imagery from Maxar. To learn more, visit https://www.mapbox.com/about/maps/ and http://www.openstreetmap.org/copyright.*

A key feature is the grouping structure table, which allows highlighting granular data levels, e.g., all species at a given site, by hiding all other data (see Figs 7 and 8). Entries in this table correspondto colored traces in the figure legend via unique numbers. Users can thus cycle through or compare deeply nested data structures. Visualizations support zooming and panning (scatter plots and maps), as well as axes scrolling and stretching on mouse hover-over. Observations can be (de-)selected through clicking or lasso and box selecting, and annotated with text labels, which are listed in a summary table below the visualization. Annotations can be provided in a text box, and are added either individually through a button click, or automatically on every selection (requires ticking corresponding box); these are added to the input data in an appended column (`.annotation`) and can be used to inform subsequent processing. Lastly, histograms of all displayed, numeric variables can be generated to assess the potential impact of data removal.

Extract recipe

Any processing (filtering, highlighting or annotating) is translated into `R` code, which can be copied or directly sent to an active `RStudio` (`R` development environment) session. The `dcr_app` can also be launched with a file path to an `*.RDS` file on disk, rather than an object in `R`'s environment. In this case, additional saving options are available for adjusting output names and file locations (Fig 4). This is currently recommended for data requiring extensive annotation, as the output script is significantly reduced. However, the option will be made available for both modes (file path, object) in a future version.

*Figure 4: **Code recipe extraction.** Options provided in the Extract tab for defining and saving outputs when `datacleanr` is launched with a file path. Copying or sending the recipe (i.e. `R` code) to an active `RStudio` session is always possible.*

Interoperability with external packages and algorithms

Interoperability is achieved with pre- and post-processing (in `R`) by two means:

1) Observations that were flagged by prior outlier or data processing have distinct symbols in interactive visualizations; this is enabled by adding (or renaming) a logical (`TRUE` / `FALSE`) flagging column named `.dcrflag` before launching `dcr_app()`.
2) The reproducible code recipe can be used as a step following or preceding additional analyses.

`datacleanr` can hence be combined with the existing strengths of `R`'s extensive ecosystem of packages to increase flexibility and, ultimately, productivity. For instance, a script-based workflow applying widely-used `R` packages for reading, "wrangling" and cleaning data, such as `readr`, `dplyr`, `tidyr` and `lubridate` from the `tidyverse` [31], as well as `janitor` [32], can

be complemented with `datacleanrs` interactivity. In addition, due to the script and file-based output, `datacleanr` can also be included in workflow management tools such as `drake` [33] and `workflowr` [34].

Efficiency and batching

`datacleanr` has been extensively tested on mobile and desktop workstations (Windows 10, Ubuntu 19.10) considered medium to high-end, and is easily capable of processing and displaying above 1 million observations simultaneously. A speed test with outlier selections at excessive and improbable scales indicated comfortable response times for most user scenarios (Fig 5).

Figure 5: ***Speed test of visualization and outlier selection on synthetic data (n = 250000).*** *Each step, 10000 (additional) points were selected (n = 25). This was repeated on low and high CPU-power settings, and processing time was determined using profvis [35]. Bands represent minimum and maximum durations, and points are means (n = 3). The inset shows processing totals (mean, min, max). Even with unlikely outlier numbers, the application remained responsive and appreciably fast.*

Nevertheless, the notable limiting factors for processing are number of columns (in exploratory summary) and the grouping structure during plotting. That is, a large number of unique groups will slow down the visualization, and we recommended aiming for a maximum of around 100 groups per `datacleanr` run.

`datacleanr::dcr_app()` returns processing results to the active `R` session. Hence, multiple datasets can be processed in batch and results (including code) saved for subsequent use. This is especially helpful when data nesting structures are too deep (e.g., ≫ 100 groups), or datasets too large (~2 million observations) to handle in one sitting. In these cases, a split-combine approach is recommended:

```r
# prepare data into species sub-sets
iris_split <- split(x = iris,
                    f = iris$Species)
# run for each species
dcr_iris <- lapply(iris_split,
                   datacleanr::dcr_app)
```

Similarly, a list of file paths to datasets can be supplied (see help in `R` via `?datacleanr::dcr_app()`).

Case studies

Below are two use-cases illustrating the utility of the interactive approach adopted in `datacleanr`.

Identifying structure and artefacts in nested data

High-volume, data with nested hierarchical structure (i.e. observations grouped at many levels) are difficult to explore and process, especially when obtained from secondary sources, where the data-generating process or the collection method are not fully known [e.g., see 36]. In such cases greater care is required, as unexpected or erroneous structures and artifacts can be present - especially so when these vary at group-level. Here, interactively engaging with the data can expedite processing while increasing confidence. As an example of dealing with such scenarios in `datacleanr`, we present a subset of nearly 320000 city and park trees listed in Berlin's (DE) green infrastructure registry (https://daten.berlin.de/, Strassenbäume, Anlagenbäume), focusing on mensuration data from the 10 most-frequent species across all 12 districts. These data were collected by different agencies or contractors (within and between districts), and are nested at multiple levels (district, street/park). For

convenient exploration with `datacleanr`, the data is grouped by district and species (Fig 6), giving 120 sub-groups.

Figure 6: ***Example set-up for hierarchically nested data.*** *Grouping structure set to district (`BEZIRK`) and species (`ART_BOT`) in "Set-up and Overview" tab for subsequent exploration, plotting and cleaning.*

Potential outliers are readily identified and annotated in a bivariate plot of tree age and diameter (Fig 7). Note, these observations could also be captured using threshold filters.

Figure 7: ***Overview of visual cleaning tab for Berlin tree data.*** *The plot shows tree age (x) and diameter (y), resolving nesting by district and species. All 120 groups are displayed (see A, and figure legend). Potential outliers are obvious and easily highlighted and annotated for later reference (B, C). The dense point cloud comprises nearly 320000 observations and requires further inspection.*

Upon cycling through the set groups, however, additional structures are apparent in the district of Neukölln for *Quercus robur* L (Fig 8), among others. These structures would only be apparent if individual visualizations (at least 120, and potentially at variable zoom) had been generated. Yet, they could not be removed easily with threshold filters and would require a high level of effort to address with manual or automated code-based processing.

Figure 8: ***Identification of problematic data structures.*** *Closer inspection of the tree dataset using the grouping structure (A) to highlight/hide specific groups. The obvious, near-perfect linear relationship between tree age and diameter at breast height requires further inspection. Concerning data points are easily selected leveraging the interactivity of the visualization (B, see inset).*

Contrastingly, with scroll and zoom in `datacleanr`, problematic observations are efficiently selected and annotated. Such observations could be erroneous, and, for example, pose an issue in hierarchical modeling, if an entire group structure is affected (e.g., random effect at

park or street level). For any subsequent removal, we take this opportunity to explicitly urge users to adhere to best practices and transparency in outlier assessment and handling [e.g., 37].

Retaining more data from time series with interactive cleaning

Time series data, e.g., from ecophysiological monitoring, can be messy due to instrument drift, response lags, power issues, etc. In high volumes, messy data may call for pragmatic decisions, such as indiscriminately removing entire periods, if interactive processing tools are not available. This may be owed to either time constraints for detailed manual processing, or because automated approaches may not accommodate unexpected processes and resulting observations. Interactive processing - both after automated quality control as well as in first instance - with `datacleanr` allows inspecting high-volume data at high resolution and to identify the impact of erroneous data points. Consequently, individual problematic observations, rather than entire periods, can be flagged and removed after careful consideration. We provide an example of manual (code-based period filtering) *vs.* interactive processing with `datacleanr` of an unpublished (in prep.) time series of raw sap flow data from the TERENO North-East Observatory [Müritz National Park, 38]. Note, in both cases due diligence and best practices were applied. With `datacleanr` more observations were retained, as individual points - not only periods - could be removed. Consequently, resulting gaps were shorter, could be gap-filled and potentially provide a higher level of insight (Fig 9). Further, the processing time decreased from approximately 2 hours by a skilled `R`-user to under 15 minutes for the entire series.

Figure 9: ***Comparison of code-based (A) and interactive processing (B) of raw sap flux data****. Interactivity allowed retaining more observations including (C, completeness by day), as compared to often more tedious, code-based filtering; additional 3 days of complete data were retained. As individual, problematic observations could be removed (D) - rather than entire periods (days, weeks) - explanatory power may be increased in subsequent analyses.*

Future developments

Development will be continued to enhance performance, and incorporate user feedback. Additional improvements are already planned and will be implemented in upcoming versions. These include: 1) saving and loading processing progress, 2) pre-select groups for plotting to reduce loading times, 3) a toggle to display filtered data (from Filter tab) in visualizations for easier assessment of filters. Further, 4) a more convenient method for gracefully handling data selections from multiple groups in the interactive visualization will be added; this is particularly helpful when, for instance, problematic observations cluster in similar plot regions.

Conclusion

Exploration and processing of high-volume data can be enhanced by using interactive tools. `datacleanr` achieves this with its flexibility and interoperability, while facilitating best practices (i.e. exploration, outlier detection, reproducibility). While we acknowledge the place for and utility of fully-automated processing pipelines, we are certain that freely-available, interactive tools will improve researchers' and analysts' necessary engagement with their data, and consequently, increase confidence in their results. Further, we believe the `datacleanr`'s design will increase productivity of both technically-proficient as well as users with limited programming ability. For this, we ensured it would fit seamlessly into existing, script-based analyses pipelines, or that it could be used as a stand-alone tool by a

wide audience. Lastly, we hope `datacleanr` will be of great use to the scientific community, including ecology, Earth system sciences and fields working with spatial and temporal data in general. We encourage users to provide feedback and suggestions in the dedicated repository to drive the continued development of the application.

Acknowledgements

We are grateful for feedback on early versions of this manuscript and the application by several anonymous individuals.

References

1. Schimel D, Keller M. Big questions, big science: Meeting the challenges of global ecology. Oecologia. 2015;177: 925–934. doi:10.1007/s00442-015-3236-3

2. Hampton SE, Strasser CA, Tewksbury JJ, Gram WK, Budden AE, Batcheller AL, et al. Big data and the future of ecology. Frontiers in Ecology and the Environment. 2013;11: 156–162. doi:10.1890/120103

3. Franklin J, Serra-Diaz JM, Syphard AD, Regan HM. Big data for forecasting the impacts of global change on plant communities. Global Ecology and Biogeography. 2017;26: 6–17. doi:10.1111/geb.12501

4. Pastorello G, Trotta C, Canfora E, Chu H, Christianson D, Cheah Y-W, et al. The FLUXNET2015 dataset and the ONEFlux processing pipeline for eddy covariance data. Scientific Data. 2020;7: 225. doi:10.1038/s41597-020-0534-3

5. Anderson-Teixeira KJ, Wang MMH, McGarvey JC, Herrmann V, Tepley AJ, Bond-Lamberty B, et al. ForC: A global database of forest carbon stocks and fluxes. Ecology. 2018;99: 1507–1507. doi:10.1002/ecy.2229

6. Kattge J, Bönisch G, Díaz S, Lavorel S, Prentice IC, Leadley P, et al. TRY plant trait database – enhanced coverage and open access. Global Change Biology. 2020;26: 119–188. doi:10.1111/gcb.14904

7. Farley SS, Dawson A, Goring SJ, Williams JW. Situating Ecology as a Big-Data Science: Current Advances, Challenges, and Solutions. BioScience. 2018;68: 563–576. doi:10.1093/biosci/biy068

8. Escamilla Molgora JM, Sedda L, Atkinson PM. Biospytial: Spatial graph-based computing for ecological Big Data. Gigascience. 2020;9. doi:10.1093/gigascience/giaa039

9. BES. A guide to data management in ecology and evolution. BES. 2014. Available: https://www.britishecologicalsociety.org/wp-content/uploads/2019/06/BES-Guide-Data-Management-2019.pdf

10. BES, Cooper N. A Guide to Reproducible Code in Ecology and Evolution. BES. 2017 [cited 23 Sep 2020]. Available: https://nhm.openrepository.com/handle/10141/622618

11. Chang WL, Grady N. NIST Big Data Interoperability Framework: Volume 1, Definitions. NIST; 2019 Oct. Report No.: 1500-1r2. Available: https://www.nist.gov/publications/nist-big-data-interoperability-framework-volume-1-definitions

12. Ward JS, Barker A. Undefined By Data: A Survey of Big Data Definitions. 20 Sep 2013 [cited 27 Jan 2021]. Available: http://arxiv.org/abs/1309.5821

13. Yenni GM, Christensen EM, Bledsoe EK, Supp SR, Diaz RM, White EP, et al. Developing a modern data workflow for regularly updated data. PLOS Biology. 2019;17: e3000125. doi:10.1371/journal.pbio.3000125

14. Zuur AF, Ieno EN, Elphick CS. A protocol for data exploration to avoid common statistical problems. Methods in Ecology and Evolution. 2010;1: 3–14. doi:10.1111/j.2041-210X.2009.00001.x

15. Benhadi-Marín J. A conceptual framework to deal with outliers in ecology. Biodivers Conserv. 2018;27: 3295–3300. doi:10.1007/s10531-018-1602-2

16. Wutzler T, Lucas-Moffat A, Migliavacca M, Knauer J, Sickel K, Šigut L, et al. Basic and extensible post-processing of eddy covariance flux data with REddyProc. Biogeosciences. 2018;15: 5015–5030. doi:10.5194/bg-15-5015-2018

17. Shaughnessy AR, Prener CG, Hasenmueller EA. An R package for correcting continuous water quality monitoring data for drift. Environ Monit Assess. 2019;191: 445. doi:10.1007/s10661-019-7586-x

18. Heer J, Kandel S. Interactive analysis of big data. XRDS. 2012;19: 50–54. doi:10.1145/2331042.2331058

19. Binnig C, Basık F, Buratti B, Cetintemel U, Chung Y, Crotty A, et al. Towards interactive data exploration. Real-time business intelligence and analytics. Springer; 2015. pp. 177–190.

20. Beilschmidt C, Drönner J, Mattig M, Schmidt M, Authmann C, Niamir A, et al. VAT: A Scientific Toolbox for Interactive Geodata Exploration. Datenbank Spektrum. 2017;17: 233–243. doi:10.1007/s13222-017-0266-5

21. Culina A, Berg I van den, Evans S, Sánchez-Tójar A. Low availability of code in ecology: A call for urgent action. PLOS Biology. 2020;18: e3000763. doi:10.1371/journal.pbio.3000763

22. R Core Team. R: A language and environment for statistical computing. Vienna, Austria: R Foundation for Statistical Computing; 2020. Available: https://www.R-project.org/

23. Rossum G. Python reference manual. Centre for Mathematics and Computer Science. 1995.

24. Gerum RC, Richter S, Fabry B, Zitterbart DP. ClickPoints: An expandable toolbox for scientific image annotation and analysis. Methods in Ecology and Evolution. 2017;8: 750–756. doi:10.1111/2041-210X.12702

25. Solsona-Berga A, Frasier KE, Baumann-Pickering S, Wiggins SM, Hildebrand JA. DetEdit: A graphical user interface for annotating and editing events detected in long-term acoustic monitoring data. PLOS Computational Biology. 2020;16: e1007598. doi:10.1371/journal.pcbi.1007598

26. Jakimow B, van der Linden S, Thiel F, Frantz D, Hostert P. Visualizing and labeling dense multi-sensor earth observation time series: The EO Time Series Viewer. Environmental Modelling & Software. 2020;125: 104631. doi:10.1016/j.envsoft.2020.104631

27. Chang W, Cheng J, Allaire J, Xie Y, McPherson J. Shiny: Web application framework for r. 2020. Available: https://CRAN.R-project.org/package=shiny

28. Sievert C. Interactive web-based data visualization with r, plotly, and shiny. Chapman and Hall/CRC; 2020. Available: https://plotly-r.com

29. Comtois D. Summarytools: Tools to quickly and neatly summarize data. 2020. Available: https://CRAN.R-project.org/package=summarytools

30. Falster DS, Duursma RA, Ishihara MI, Barneche DR, FitzJohn RG, Vårhammar A, et al. BAAD: A Biomass And Allometry Database for woody plants. Ecology. 2015;96: 1445–1445. doi:10.1890/14-1889.1

31. Wickham H, Averick M, Bryan J, Chang W, McGowan LD, François R, et al. Welcome to the tidyverse. Journal of Open Source Software. 2019;4: 1686. doi:10.21105/joss.01686

32. Firke S. Janitor: Simple tools for examining and cleaning dirty data. 2020. Available: https://CRAN.R-project.org/package=janitor

33. Landau WM. The drake R package: A pipeline toolkit for reproducibility and high-performance computing. Journal of Open Source Software. 2018;3. Available: https://doi.org/10.21105/joss.00550

34. Blischak JD, Carbonetto P, Stephens M. Creating and sharing reproducible research code the workflowr way [version 1; peer review: 3 approved]. F1000Research. 2019;8. doi:10.12688/f1000research.20843.1

35. Chang W, Luraschi J, Mastny T. Profvis: Interactive visualizations for profiling r code. 2019. Available: https://CRAN.R-project.org/package=profvis

36. García Criado M, Myers-Smith I, Baeten L, Cunliffe A, Daskalova G, Gallois E, et al. Sharing is Caring: Working With Other People's Data. methods.blog; 4 Sep 2020 [cited 24 Sep 2020]. Available: https://methodsblog.com/2020/09/04/sharing-is-caring-working-with-other-peoples-data/

37. Aguinis H, Gottfredson RK, Joo H. Best-Practice Recommendations for Defining, Identifying, and Handling Outliers. Organizational Research Methods. 2013;16: 270–301. doi:10.1177/1094428112470848

38. Heinrich I, Balanzategui D, Bens O, Blasch G, Blume T, Böttcher F, et al. Interdisciplinary geo-ecological research across time scales in the northeast german lowland observatory (TERENO-NE). Vadose Zone Journal. 2018;17: 1–25.

Colophon

This report was generated on 2021-02-19 16:04:24 using the following computational environment and dependencies:

```
#> - Session info ---------------------------------------------------------------
----
#>  setting  value
#>  version  R version 3.6.3 (2020-02-29)
#>  os       Windows 10 x64
#>  system   x86_64, mingw32
#>  ui       RTerm
#>  language (EN)
#>  collate  English_United States.1252
#>  ctype    English_United States.1252
#>  tz       Europe/Berlin
#>  date     2021-02-19
#>
#> - Packages -------------------------------------------------------------------
----
#>  package     * version date       lib source
#>  assertthat    0.2.1   2019-03-21 [1] CRAN (R 3.6.2)
#>  bookdown      0.21    2020-10-13 [1] CRAN (R 3.6.3)
#>  callr         3.5.1   2020-10-13 [1] CRAN (R 3.6.3)
#>  cli           2.2.0   2020-11-20 [1] CRAN (R 3.6.3)
#>  crayon        1.3.4   2017-09-16 [1] CRAN (R 3.6.2)
#>  desc          1.2.0   2018-05-01 [1] CRAN (R 3.6.2)
#>  devtools      2.3.2   2020-09-18 [1] CRAN (R 3.6.3)
#>  digest        0.6.27  2020-10-24 [1] CRAN (R 3.6.3)
#>  ellipsis      0.3.1   2020-05-15 [1] CRAN (R 3.6.3)
#>  evaluate      0.14    2019-05-28 [1] CRAN (R 3.6.2)
#>  fansi         0.4.1   2020-01-08 [1] CRAN (R 3.6.2)
#>  fs            1.5.0   2020-07-31 [1] CRAN (R 3.6.3)
#>  glue          1.4.2   2020-08-27 [1] CRAN (R 3.6.3)
#>  htmltools     0.5.0   2020-06-16 [1] CRAN (R 3.6.3)
#>  knitr         1.29    2020-06-23 [1] CRAN (R 3.6.3)
#>  magrittr      2.0.1   2020-11-17 [1] CRAN (R 3.6.3)
#>  memoise       1.1.0   2017-04-21 [1] CRAN (R 3.6.2)
#>  pkgbuild      1.1.0   2020-07-13 [1] CRAN (R 3.6.3)
#>  pkgload       1.1.0   2020-05-29 [1] CRAN (R 3.6.3)
#>  prettyunits   1.1.1   2020-01-24 [1] CRAN (R 3.6.2)
#>  processx      3.4.5   2020-11-30 [1] CRAN (R 3.6.3)
#>  ps            1.5.0   2020-12-05 [1] CRAN (R 3.6.3)
#>  R6            2.5.0   2020-10-28 [1] CRAN (R 3.6.3)
#>  remotes       2.2.0   2020-07-21 [1] CRAN (R 3.6.3)
#>  rlang         0.4.9   2020-11-26 [1] CRAN (R 3.6.3)
#>  rmarkdown     2.6     2020-12-14 [1] CRAN (R 3.6.3)
#>  rprojroot     2.0.2   2020-11-15 [1] CRAN (R 3.6.3)
```

```
#>  sessioninfo   1.1.1   2018-11-05 [1] CRAN (R 3.6.2)
#>  stringi       1.5.3   2020-09-09 [1] CRAN (R 3.6.3)
#>  stringr       1.4.0   2019-02-10 [1] CRAN (R 3.6.2)
#>  testthat      3.0.0   2020-10-31 [1] CRAN (R 3.6.3)
#>  usethis       1.6.3   2020-09-17 [1] CRAN (R 3.6.3)
#>  withr         2.3.0   2020-09-22 [1] CRAN (R 3.6.3)
#>  xfun          0.17    2020-09-09 [1] CRAN (R 3.6.3)
#>  yaml          2.2.1   2020-02-01 [1] CRAN (R 3.6.2)
#>
#> [1] C:/Program Files/R/R-3.6.3/library
```

The current Git commit details are:

```
#> Local:    master C:/Users/ahurl/Documents/_work/p035_datacleanr/dcrpaper
#> Remote:   master @ origin (https://github.com/the-Hull/dcrpaper.git)
#> Head:     [f7fa268] 2021-02-17: formatting
```

Conceptual workflow

Click here to access/download;Figure;Fig1.tif

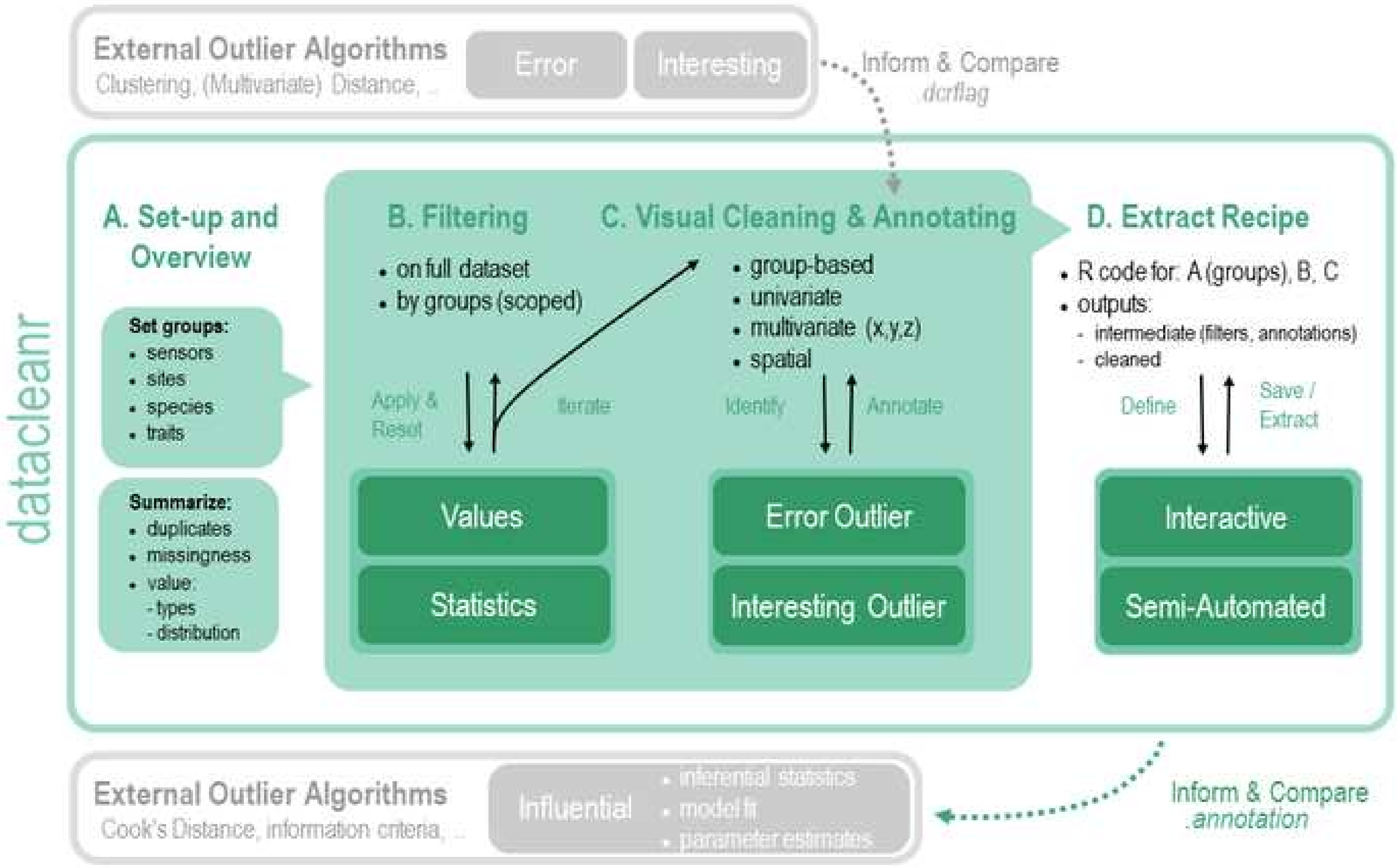

Click here to access/download;Figure;Fig2.tif

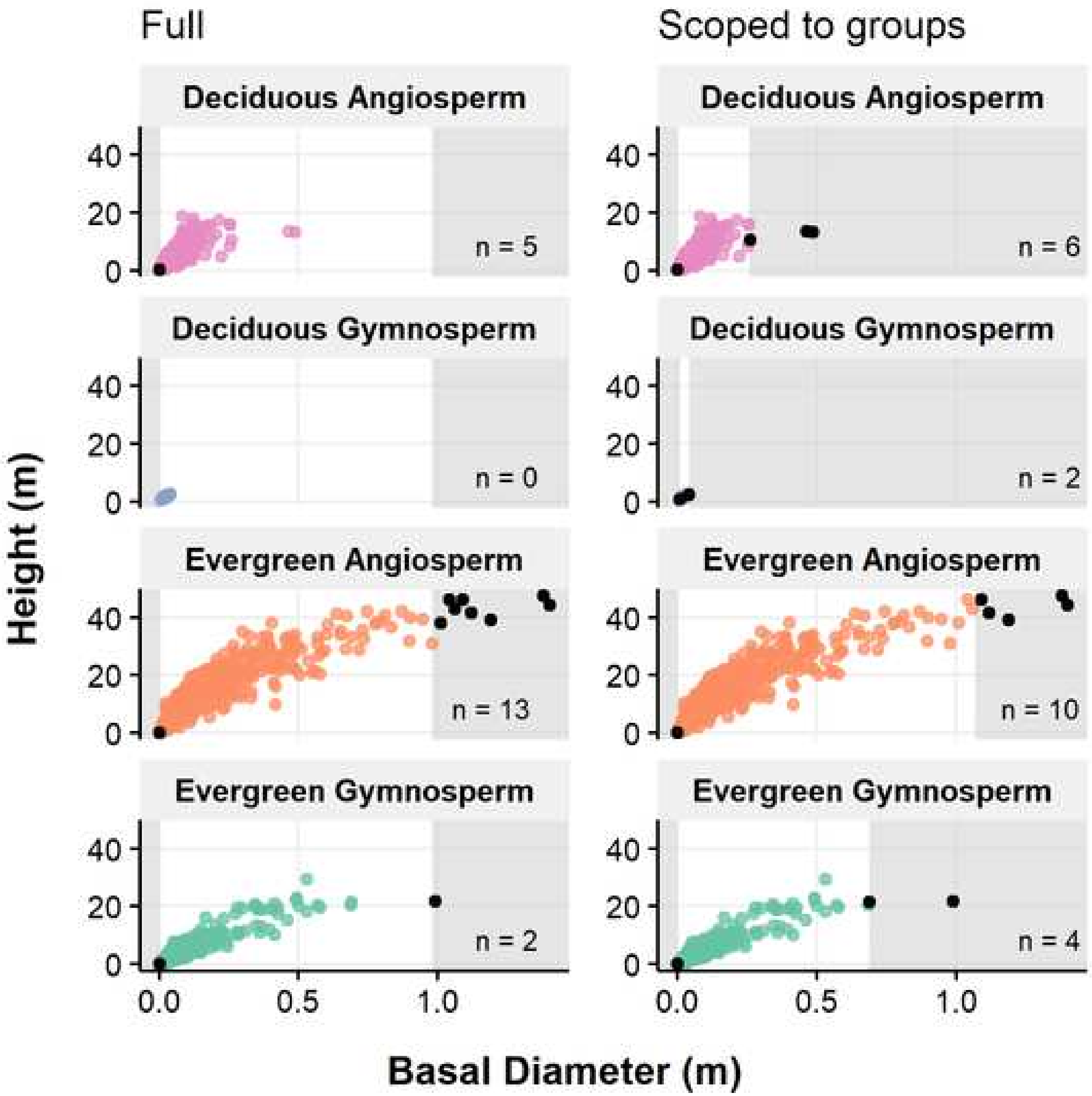

Click here to access/download;Figure;Fig3.tif

Background

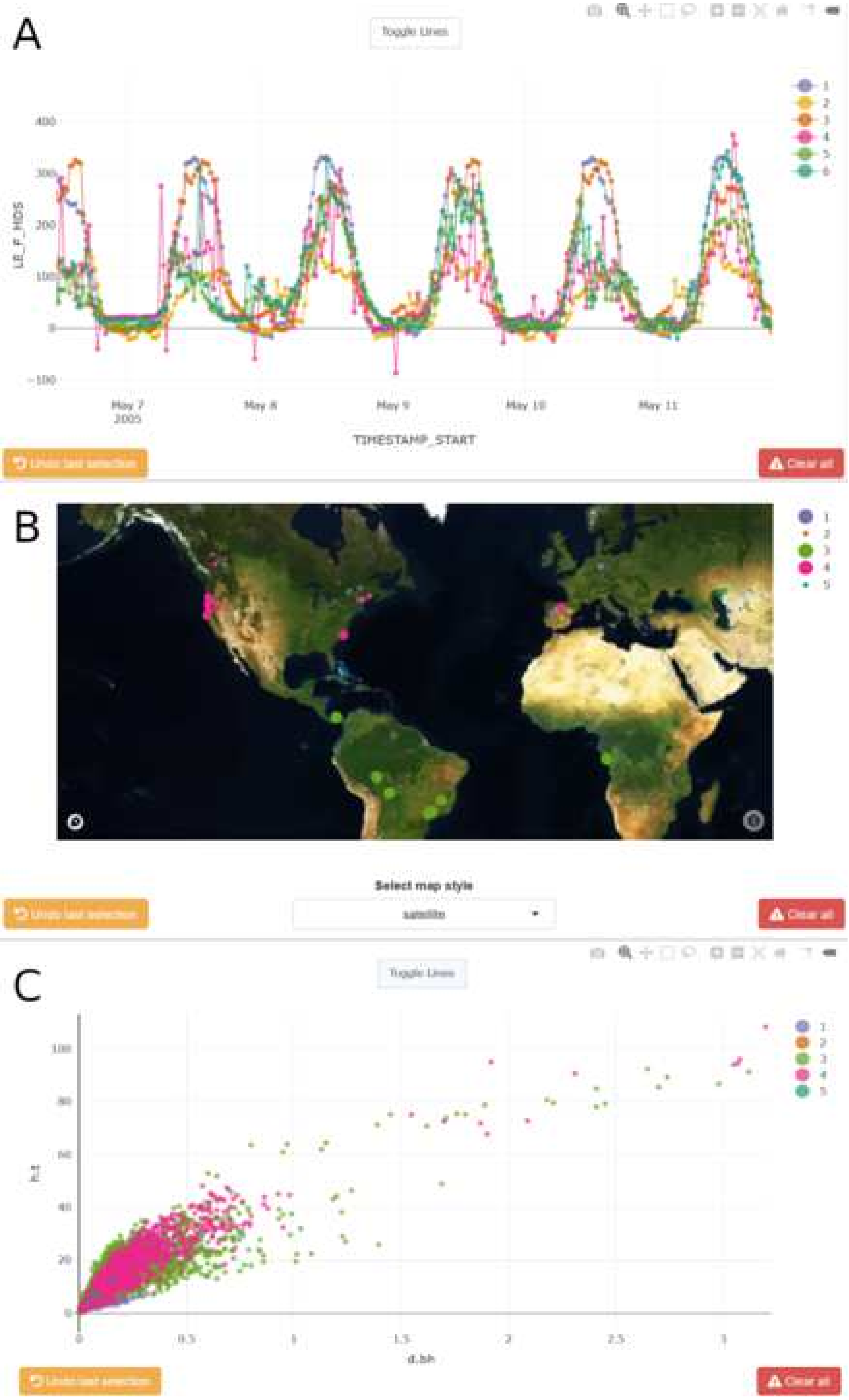

Extract tab

Click here to access/download;Figure;Fig4.tif

Pasted Layer #1

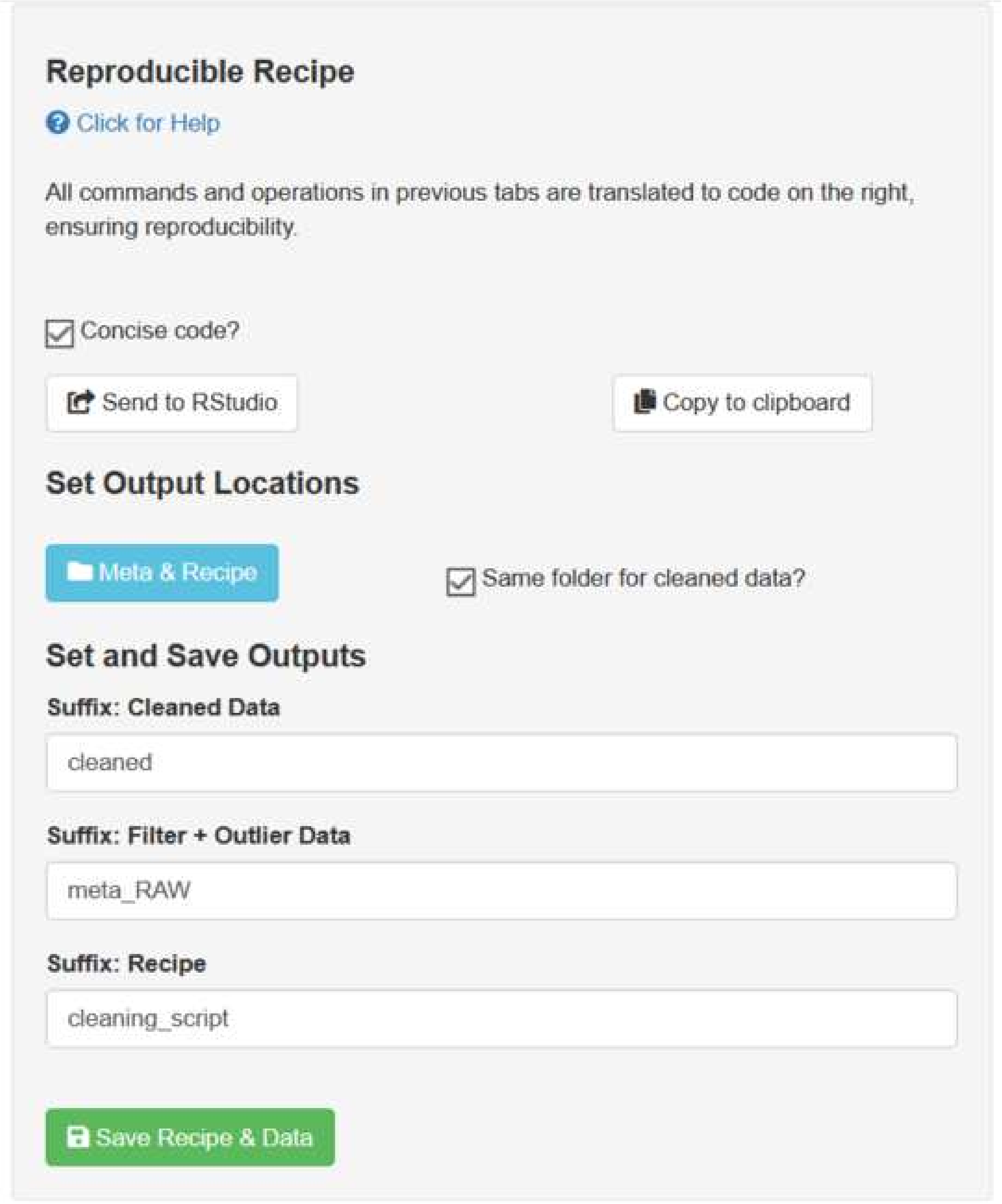

 Click here to access/download;Figure;Fig5.tif

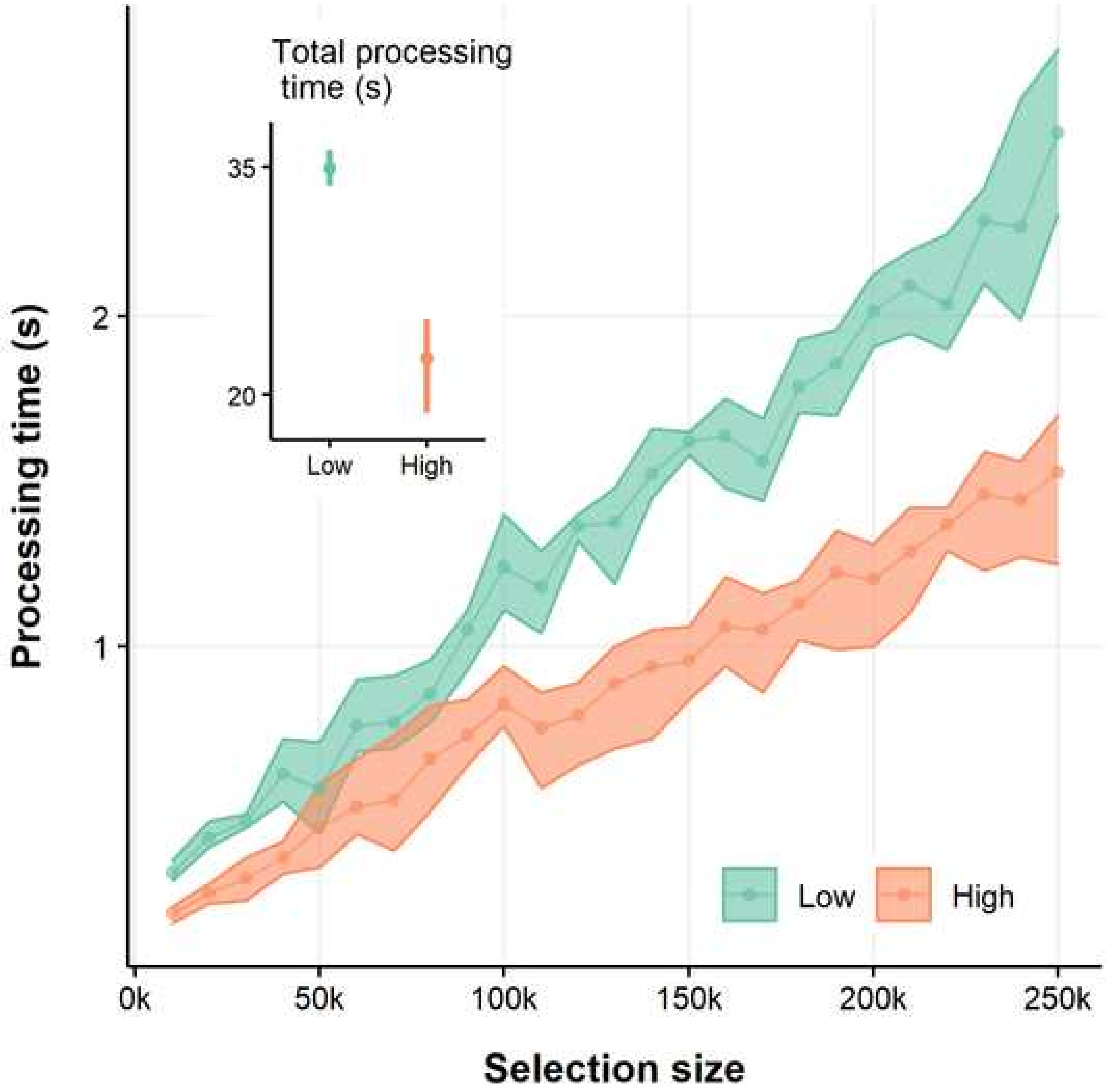

Grouping set-up

Click here to access/download;Figure;Fig6.tif

Background

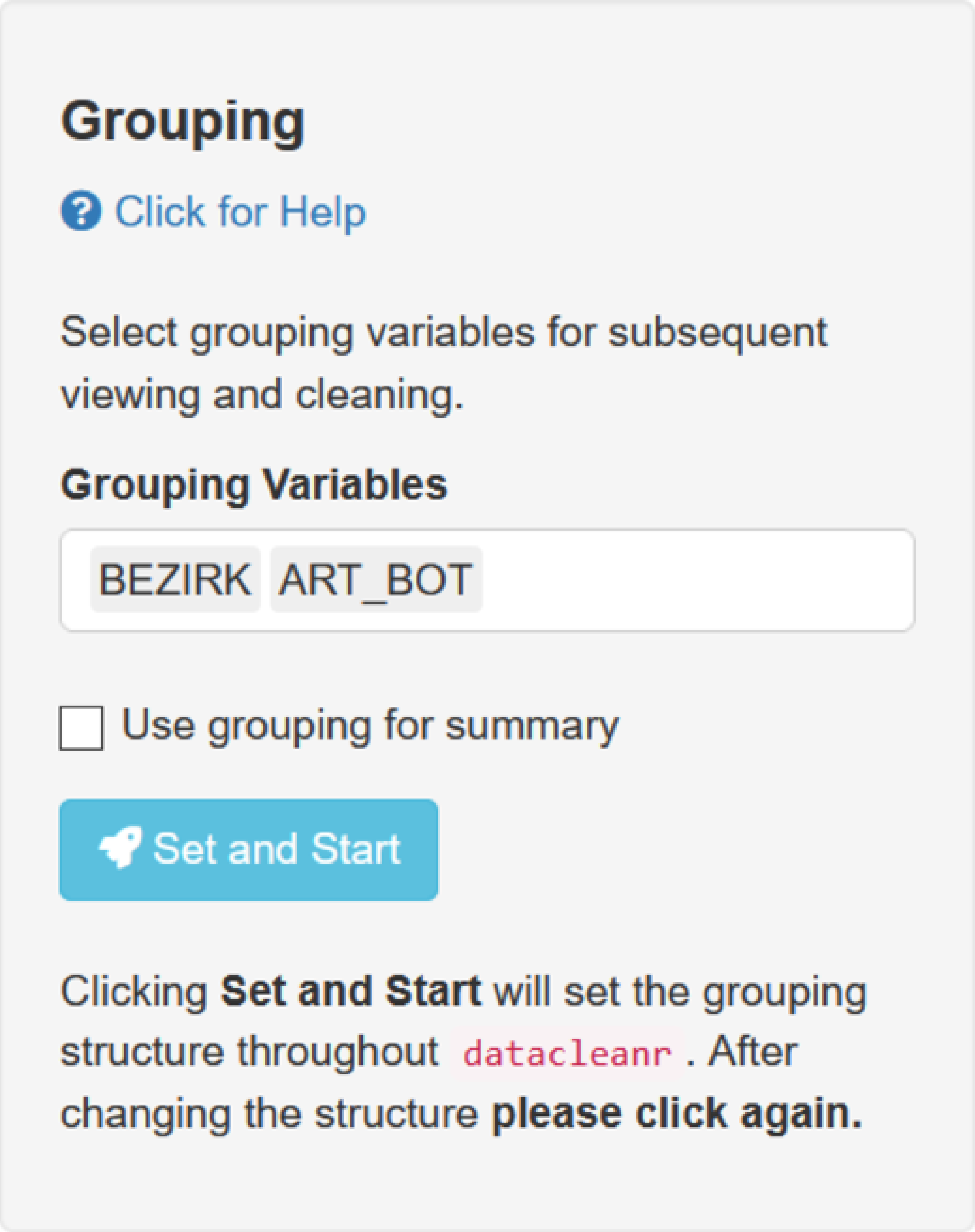

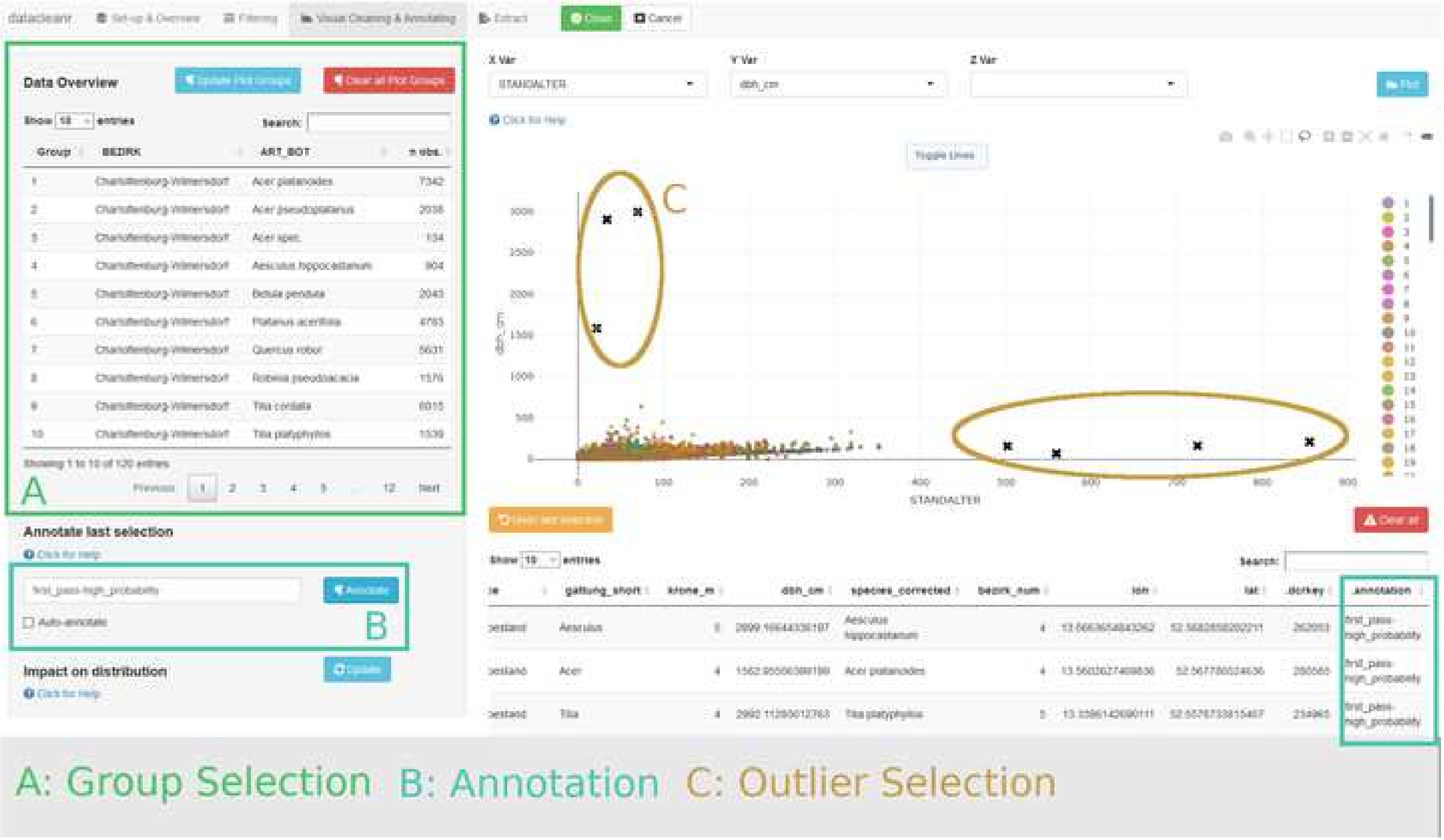
Data Overview
Group
BEDRK
ART_BOT
n obs.
Annotate last selection
Impact on distribution
X Var
Y Var
Z Var
STANDALTER
A
B
C
A: Group Selection B: Annotation C: Outlier Selection

Data exploration zoomed

Click here to access/download;Figure;Fig8.tif

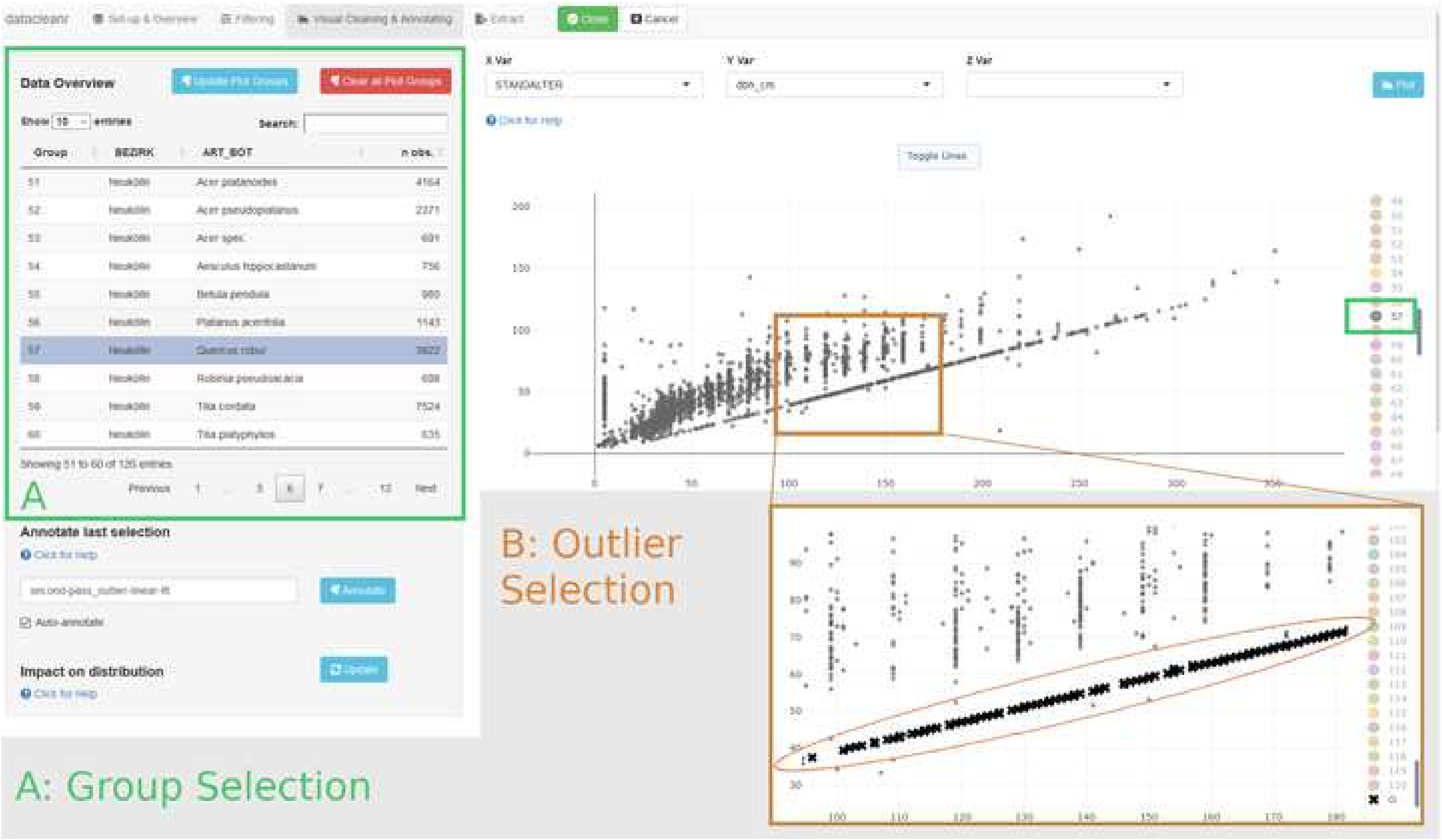

Click here to access/download;Figure;Fig9.tif

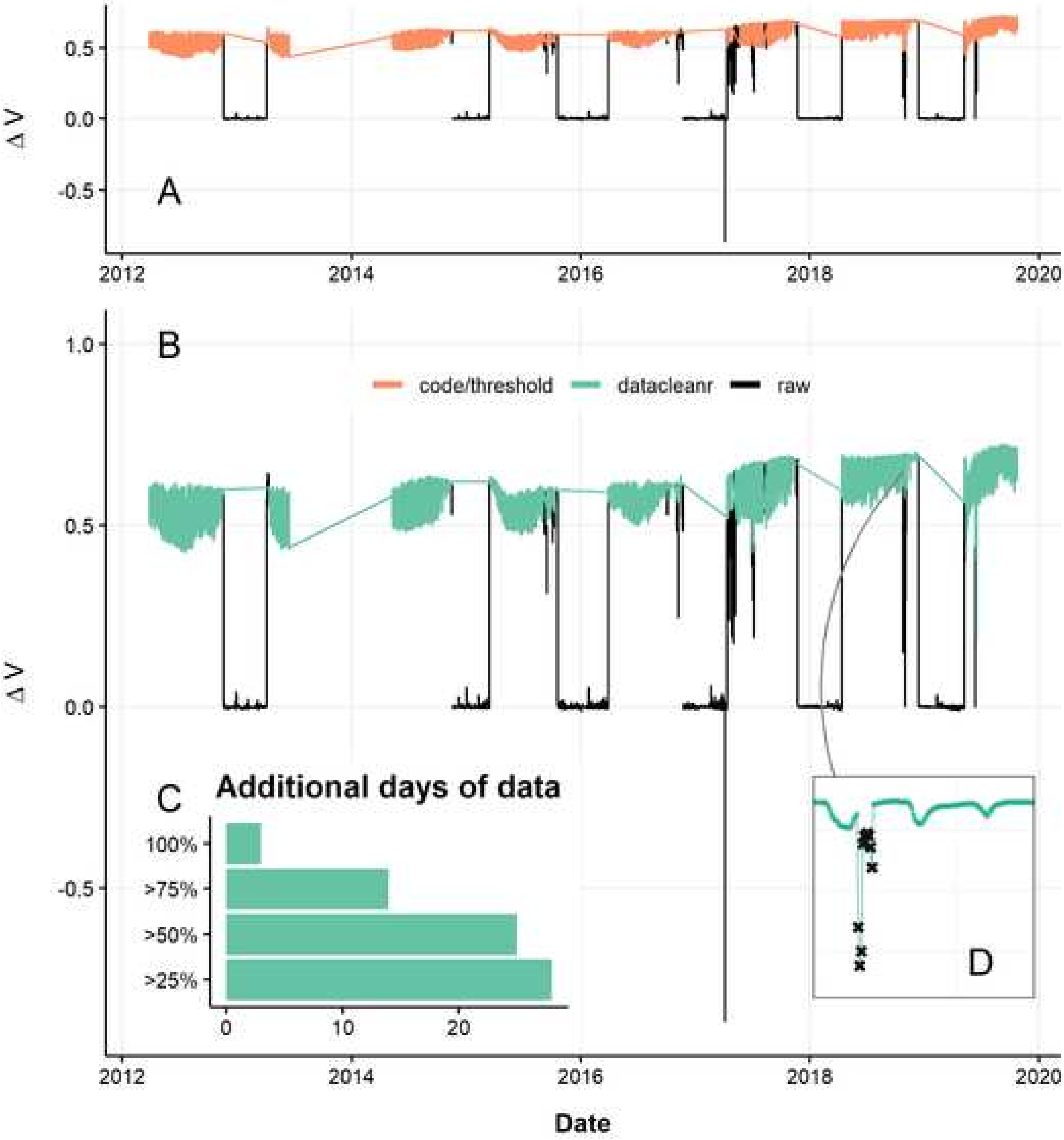

Supplement: Attachment — Submitted filename: PONE-D-21-05607_reviewer.pdf [file pone.0268426.s002.pdf]
