# Supplementary material for: Addressing the need for interactive, efficient, and reproducible data processing in ecology with the datacleanr R package
Source: PLoS One. 2022 May 12;17(5):e0268426. doi: 10.1371/journal.pone.0268426 (PMC9098071; doi:10.1371/journal.pone.0268426)
Supplement: Attachment — Submitted filename: Response to Reviewers.docx [file pone.0268426.s003.docx]

Addressing the need for interactive, efficient, and reproducible data processing in ecology with the datacleanr R package

# Response to reviewers

## Editor

The reviewers raised a number of concerns with your study, in particular the ease of use of the program and the lack of a walk-through analysis, as well as some points to improve clarity. Their comments can be viewed in full, below and in the attached file.

We thank the editor and reviewers for their time and the opportunity to improve the manuscript and thus the (initial) user experience of datacleanr. We have carefully considered the concerns raised by the referees and included a detailed walk-through analysis to further enhance the usability of the presented package. With these suggestions we feel that the manuscript has significantly improved.

## Reviewer 1

The manuscript introduces an R package for data processing of large ecological data sets. It gives an overview of the package’s functionality. I think that such an R package is useful, especially for the scenarios the authors have pointed out (large data sets with many different scales / scopes, large monitoring data sets), for quick visualizations, outlier and problem detection.

We thank the reviewer for their assessment and recognition of our tool’s utility.

I only have a few suggestions for improving the manuscript:

We have addressed all comments by implementing the suggested changes or by providing a justification for the issue at hand in its current state. An overview thereof is given below.

*1. line 139 Figure 1 caption: Sometimes the authors refer to specific functionality that the reader would only really understand when running the package, e.g. line 149 after “Set and Start” is clicked. This detail is perhaps too technical for this overview.

We improved the wording of technical details here, enhanced Fig 1 caption (package overview, L165 in response) to better guide the reader, and added a SI File with animated examples for a walk-through, as also suggested by Reviewer 2. The section mentioned above now reads:

“The structure is available during targeted filtering (scoping; see *Filtering*) and visual cleaning (see Figs6-8). Once the grouping is set, a dataset summary can be generated via the package summarytools [29], highlighting duplicates, missingness, and distribution of each variable.”

As noted above, the caption of Fig 1 has been improved significantly, in accordance with Reviewer 2’s suggestions (see in respective section further below, L165), which we believe allows the reader to follow the descriptions more readily.

*2. line 151 via 29 …. would be more useful if it named the package directly

Adjusted to “..summary can be generated via the package summarytools [29]..”

*3. line 156: not sure what ‘through text cues on the tab’ refers to

The section in question now reads:

“The application’s interactivity allows reviewing the impact of filters through a text note highlighting the percentage of removed data and an overview table showing the remaining observations (per group), …”

*4. Figure 2 and caption: To me it is not entirely clear what is displayed here. E.g. what is the difference between left and right, what the n = .. refer to? Make clearer that the grey areas correspond to points which are filtered out?’

We appreciate the caption was not detailed enough and have adjusted it to:

“Figure 2: Example of the impact of statistical filtering on bivariate relationships between trait data from BAAD [30]. A percentile threshold filter (0.01 and 0.99) is used to remove extreme low and high values on the x-variable across its full space (left) or scoped to groups represented by functional types (right). The gray shading indicates the filtered variable space (full or scoped), while text labels and black points count and highlight, respectively, individual observations captured by the applied filter. Note, the figure was not generated in datacleanr.”

We are confident that the adjusted caption sufficiently explains the figure and highlights the difference between full and scoped filtering adequately.

*5. line 167 — ‘any statement’: make clearer that this refers to the filtering statement, it would be useful to add that this filtering statement requires R code

The text was adjusted to:

“Any filtering statement (provided as valid R code) which evaluates to TRUE or FALSE..”

*6. line 169: insert ‘the’ before ‘following’

Done.

*7. line 185: more useful to refer directly to ‘plotly’

Done.

*8. line 186: insert ‘be’ before ‘displayed’

Done.

*9. line 197: clarify what ‘key feature’ refers to. I think it refers to the visualization tab, but this is not clear.

This was changed to:

“A key feature on the visualization tab is the grouping structure table…”

*10. line 199: correspond to

Done.

*11. line 242, Figure 5 caption: not sure what ‘processing totals’ refer to. Why (n=3) after means? Does this imply that there were 3 runs at every setting (number of points)?

The caption was adjusted to:

“Figure 5: Speed test of visualization and outlier selection on synthetic data (n = 250000). In 25 consecutive steps 10000 (additional) points were selected. This was repeated three times on low and high CPU-power settings, and processing time was determined using profvis [35]. Bands represent minimum and maximum durations, and points are means of the three replicates. The inset shows processing totals (mean, min, max) after completing all 25 selections. Even with unlikely outlier numbers, the application remained responsive and appreciably fast.”

*12. line 270: remove comma after high-volume

Done.

*13. Figure 9 and caption (line 331): I cannot see a difference between figures A and B. Therefore, is A necessary. If there are differences, maybe highlight these or point out. Also, I am not sure how to understand panel C. ‘including’ in caption misplaced?

We appreciate this issue, and have previously tried different versions of the figure with overplotting and offsetting in a single panel. However, neither option was fully satisfactory. This was either due to the same issue (distance between lines) or overplotting due to the time scale. We do want to emphasize the entire time series to highlight the package’s capability of dealing with fairly large/high-resolution data here for one processing example, and have used the caption to better highlight visible differences, , although these admittedly require increased attention. The caption now reads:

“Figure 9: Comparison of code-based (A) and interactive processing (B) of raw sap flux data. Compared to often more tedious, code-based filtering, interactive quality control using domain expertise allowed retaining more observations resulting in greater data coverage. Here, three additional full days of measurements, as well as several days with varying partial coverage were retained (C, completeness by day; e.g. 25 % of all measurements for a given day). For example, differences are found in 2014 and 2017, where the code-based filtering removes entire periods (i.e., days, weeks). By contrast, individual, problematic observations could be removed interactively (D), which may increase explanatory power in subsequent analyses.”

## Reviewer 2

It has one extremely important feature, i.e. it returns the R code for doing the filtering and the cleaning of the data, i.e. therefore making it transparent and reproducible. This is an essential feature, which bridges the gap between purely code based outlier removal and interactive outlier identification.

We thank the reviewer for the recognition of datacleanr’s utility and the thorough review.

But as the R script is effectively a script adding a column identifying if a data point was identified as an outlier or not, it would be very useful to also generate a report which includes e.g. the graphs in which the outliers were identified and the filtering rules as an html or pdf which can be added to the data as a justification why the points were identified as outliers.

We discussed similar features for PDF or HTML reports during datacleanr’s development. We agree that a justification or annotation for conspicuous data is not only useful but necessary. This is why we implemented the annotation feature in the interactive data selection. This gives users the freedom to use a set of self-defined annotations or tags for different scenarios (e.g., “high value,” “battery failure,” etc.). These annotations are stored in the .annotation column, and allow to not only identify selected points in a boolean manner, but also provide the user-specified annotation. From our own use of datacleanr we have concluded that this approach affords flexibility down the line to implement case-specific solutions, such as bespoke graphs and tables, which can be included in user-specific outputs (e.g., PDF or HTML reports based on RMarkdown). We also would like to emphasize that the reproducible recipe provides all the necessary information for such reports - or even as a stand-alone overview - if the data selection and annotation is done with due diligence.

In the ideal case (future development?) I would suggest a config file (yaml?) which contains all the info and settings used, and when loaded, loads the data and applies all settings from the previous session.

This is most certainly planned as a future development, and will likely rely on the new shiny caching feature, rather than a yaml config file – we will explore the latter option as well and appreciate the idea/pointer, however. As we recognize the importance of this feature it is also the first we mention in this section (with updated wording):

“Additional improvements are planned and will be implemented in upcoming versions. These include: 1) saving and loading processing progress within the application …”

Unfortunately, I was only able to test the app after quite a bit of trying, as no walk-through of the data analysis is provided. This is a pity, as all the data is available in appropriate licenses and all that would be needed is to supplement the manuscript with boxes showing the settings for each step. This relatively easy addition would make the manuscript much more approachable.

Thank you for highlighting this. We have included the package’s readme file (https://github.com/the-Hull/datacleanr or on CRAN as https://cran.r-project.org/web/packages/datacleanr/readme/README.html) with animated examples as an SI file (modified to comply with the 20 MB file restriction), which we consider even more instructive than a walk-through with screenshots only. It includes details on installation, capabilities, and use, with in-depth examples of every feature. We note this in the “Capabilities section” to prime the reader. The paragraph now reads:

“datacleanr is an interactive R package for processing high-volume data, and it caters to best practices in data exploration, processing, and reproducibility. This section describes the general capabilities of the package, and an in-depth walk-through of all functionalities is provided with animated examples in S1 File).”

Overall, I have added a number of comments to the pdf document (attached) which can be easily adressed.

Thank you for the thorough comments. We have adjusted the text accordingly.

A list of noteworthy alterations or responses to comments in the PDF beyond simple text adjustments:

- the title of the article was changed to read “package” rather than “application,” and is referred to as such throughout the article now
- added DOI (<https://doi.org/10.5281/zenodo.6337609>) and version number (v1.0.3) to “Availability” section
- Updated data availability statement to include “latest version” Zenodo DOI, for which the archive now contains CSV data only: <https://doi.org/10.5281/zenodo.4550726>
- instead of noting an explicit example with respect to financially inaccessible software, we rephrased to “..if convenient tools do not exist or are financially inaccessible due to commercial licensing”
- tibbles can be supplied to dcr_app(); the help documentation for dcr_app() notes that the data can be a data.frame, tbl (tibble), or data.table
- The caption for Figure 1 was modified to address all processing modules. It now reads: *Figure 1: Conceptual workflow for datacleanr across its four processing modules. A) The Set-up and Overview tab allows for a quick initial assessment of a data set (variable types, distribution, completeness), where nested structures (e.g., by plot, site, region) can be resolved by defining a grouping structure from a categorical data column. B) The Filtering tab allows sub-setting the data based on valid R code (logical statements), which can be targeted (i.e., “scoped”) to individual groups from A). C) The Visual Cleaning and Annotating tab allows generating two or three dimensional visualizations (X, Y, and point size) rapidly, while dividing the data set into groups specified in A); data points for further inspection can be identified by clicking or lasso selection through which annotations may also be added. An overview table and histogram highlight selected points and the potential impact on the data’s distribution should the selected observations be removed. D) The Extract recipe tab generates code to reproduce all processing steps, which can be copied to the clipboard or sent directly to an active RStudio session’s script; depending on the processing mode (in memory or from a file), additional settings for file name specification are available. The schematic here illustrates the potential for including datacleanr into an existing workflow, for example, with prior determination of outliers using external algorithms (requires appending a logical TRUE/FALSE column named .dcrflag), interactive exploration and processing (with datacleanr), and informing subsequent analyses by drawing on the interactively annotated data (.annotation column in output from datacleanr).*
- We considered your comment on using text-based data objects, as opposed to binary files. We strongly feel that the binary format for use with datacleanr is necessary, as this is the only way to ensure that data input/output does not alter data types, for example from factor or time to character, which may require that additional bespoke code is added to the extracted recipe. We cannot ensure that this is would be done correctly during generation of the recipe, and thus prefer the *.RDS format. We appreciate the limitation on universal data input/output, however.
- *Is the cleaning (or any other aspects in this app) paralellised, and could you gain a substantial increase if you do this?*
  Currently, the data selection (point clicking and lasso selection) is implemented by carrying point indices and plotly trace numbers (i.e., group numbers) in a data.frame. The limiting factors are checking for duplicates and redrawing the “outlier” trace (which is plotly’s equivalent of a ggplot2 geom). In fact, due to plotlys mechanics, the entire plot needs to be redrawn or refreshed when traces are manipulated. Packages such as multidplyr may be interesting for subsetting individual groups in the Filtering tab, but those operations are certainly not a bottleneck in processing time. We do, however, strive to enhance the user experience in the future by further decreasing processing time with streamlining above mentioned data.frame operations.
- We appreciate you highlighting the necessity to provide information on why observations were considered conspicuous, erroneous or outliers, and have rephrased line 310 (PDF) to: *We take this opportunity to explicitly urge users to make extensive use of the annotation feature on the Visualization tab to provide rich information on the selected observations (e.g., “interpolated observations”), as well as to adhere to best practices and transparency in outlier assessment and handling [e.g., 37] for any subsequent removal.*
- Comments on future developments were:
  - maybe included but not mentioned here: generate report which can include comments to why certain decisions were taken -THIS WOULD BE A VERY USEFUL FEATURE. *See previous comments on annotation tool and flexibility (L111 in response)*
  - loading from other file formats csv, txt, databases (DBI). *See previous comments on file formats; database connections were data types are preserved could be a viable option, however, which we will consider for future developments*
  - parallelisation of processing*. See previous comments (L199 in response)*
  - include splicing in the app (probably loading data into an sqlite database and query subsets out)? *We have included this in the list of future developments within the text (L401 in Revised Mansuscript with Track Changes.docx)*
  - I don’t know if it is possible - reduce the dependencies. We have done our best to keep dependencies as low as possible, but shiny and plotly are rather heavy. However, we still strive to drop dependencies where possible, and will aim to do so in the future, e.g., for new developments.
  - add vignettes to the package (possibly this paper? License?). *We appreciate the utility of vignettes. However, the GitHub repository (*[*https://github.com/the-Hull/datacleanr*](https://github.com/the-Hull/datacleanr)*) has an extensive set of examples with GIFs, which we believe are better suited to highlight the package’s functionality – this now constitutes the walk-through in the supporting information in a somewhat reduced fashion to meet the <20 MB file size requirements We also would like to note that CRAN very regularly enforces a <5 MB package size policy, and believe the GIFs in the ReadMe do better justice to the packages functionalities than a screenshot-based document of smaller size. The license for the package is GPL-3 and listed in the Availablility section.*
- In response to the comment on enhancement of data analyses through interactivity, we rephrased the first sentences of the conclusion to: “Exploration and processing of high-volume data can be enhanced by using interactive tools. datacleanr achieves this with its flexibility and interoperability, while facilitating best practices in data exploration, outlier detection, and especially reproducibility through the extractable code recipe.”

As mentioned, my main concern is the missing of a walk-through through one analysis. I would suggest that this relatively straight forward addition / change is done before publication.

We appreciate this concern and are confident our animated examples in the Supporting Information file S1 File (Walkthrough.html) sufficiently address this concern.
