# Supplementary material for: Addressing the need for interactive, efficient, and reproducible data processing in ecology with the datacleanr R package
Source: PLoS One. 2022 May 12;17(5):e0268426. doi: 10.1371/journal.pone.0268426 (PMC9098071; doi:10.1371/journal.pone.0268426)
Supplement: Attachment — Submitted filename: Response to Reviewers.docx [file pone.0268426.s004.docx]

Addressing the need for interactive, efficient, and reproducible data processing in ecology with the datacleanr R package

# Response to Editor

## Editor Comments

Thanks a lot for addressing all points raised by the reviewers. I have only a few minor points which should be adressed before publication and some more general comments.

We thank the editor for their time (also for the previous review) and the opportunity to further improve the manuscript. We have carefully considered the concerns and are confident that we fully address them in this second round of revisions. Below are responses to specific comments. Note that line references refer to Revised *Manuscript with Track Changes.docx*

### Text:

- Please include a reference for RStudio, as it is done for e.g. R
  - Done. See Figure 1 and 3 Captions, as well as main text (L168, L260, L273)
- Figure 2 caption: clarify what n means
  - The caption was adjusted to read:
    “… where the gray shading indicates the filtered variable space and text labels count the excluded observations (e.g., ‘n = 2’).” (L207)
- Sort figs according to their first occurrence in the manuscript (i.e. 6-8 after fig 1)
  - We adjusted this cross reference to note sections, rather than figures, to maintain the current figure order by rephrasing to:
    “visual cleaning (see section Visual cleaning and annotating, as well as Case studies).” (L180)
- Availability Section: Start with stating which version you are using in the package and mention the DOI - than mention stable release and where one can get these from (DOI to newest version, 10.5281/zenodo.6337608, CRAN) and finally github repo as newest version. Probably one sentence about release plans to CRAN (always latest stable), github master / main is stable? Do you have a dev branch for development and possibly unstable versions?
  - This section now reads:
    “This publication used v1.0.3 of datacleanr, which is permanently archived on Zenodo under https://doi.org/10.5281/zenodo.6337609. Stable package releases are available on the Comprehensive R Archive Network (CRAN; use install.packages(“datacleanr”)), which aim to mirror new developments provided and documented on a dedicated repository (www.github.com/the-hull/datacleanr). The repository provides installation instructions for all sources (CRAN, repository) and animated demonstrations with test data. datacleanr is available under a GPL-3 license.”
    We do not yet use a dev branch on the repository, but may consider so for new developments and especially when issues are reported for transparency as well as interactive and user-base driven development. (L125 and beyond)
- Capabilities l 135 add "in the supplemental material" or "LINK TO THE PERMANENT FILE IN GITHUB"
  - Adjusted the text to the suggested “..animated examples in S1 File in the supplemental material.” (L140)
- l 238: "2 million observations smoothly" - is this a hard link, or soft link, after which it get's slower but still work? Also, in line 270 you mention one milion.
  - Thank you for noting this. The visualizations still work, but slow down dramatically, depending on computing power. This is not a hard limit, and based on user and dev reports in online forums.
    The text now reads:
    “where modern browser capabilities allow displaying approximately 2 million observations smoothly, around which the visualizations and processing increasingly slow down (dependent on computing power)”. (L143)
- I stil think it would be nice to have screenshots in the sections "Capabilities" when you refer to the individual steps (you have them in the visualization section), but I appreciate your point that the animated gifs give a better point. Can you put direct links to the animated gifs in the section they refer to and keep these permanent (e.g. link to a specific tagged release on github)?
  - I added the “key” GIFs to Zenodo individually to generate DOIs and ensure file permanence; DOI links to these GIFs are now provided at/near the beginning of every respective Capabilities section.
    For example:
    “Filtering (Fig1, B; also see animation at https://doi.org/10.5281/zenodo.6469721) is done by adding filter statement text boxes by clicking on the respective button on the tab.” (L178, L185, L227, L260)
- l 264: Add individual references to the individual packages (readr, dplyr, tidyr and lubridate)
  - Done. (L284)

### Figures:

- Figure 5: with three replicates, to plot mean,min and max (which are two of the three points) is an aggressive approach. I would rather leave the shaded area and plot, instead of the mean, the third point. I do not see it as necessary as in this context, as the graph is not at all crucial to the paper, but regard would regard it as a cleaner approach.
  - Thank you for this suggestion. The figure has been altered to show all three measurements along with the band as a visual aid.
    The caption now reads (L296):
    “**Speed test of visualization and data selection on synthetic data (n = 250000)**. In 25 consecutive steps 10000 (additional) observations were selected. This was repeated three times (points) on low and high CPU-power settings, and processing time was determined using profvis [40], with bands plotted as visual aids. The inset shows processing totals (mean, min, max) after completing all 25 selections. Even with selections representing unlikely outlier numbers, the application remained responsive and appreciably fast.”

- Figure 9 caption: re comment reviewer 1 on difference: I agree - I still, although explained in the caption, struggle to see where there is a difference. Also, I think the actual values of deltaV are not relevant in this graph. I think as a y asis, you would simply need four categories (from top to bottom): data in original data, day removed by using code based filering, day removed by using interactive processing, days gained by using interactive processing. These would be the relevant info, as the values at these days are not relevant based on the caption. If you want to retain the graph as it is, I would strongly suggest to have a fourth (or fifth?) colour, which highlights the data points retained in addition to the code based filtering.
  - We appreciate this suggestion, as we have iterated this graph several times. Based on this, we present a version with slight modification from your initial suggestion, as we do want to highlight not only complete days of data, but also individual days where only few observations are missing. We hope to achieve this by focusing on the previous sub-panel, highlighting data completeness by day, for both code-based/threshold and datacleanr-based processing. The difference between both methods is noted by text labels, as the x-axis range would otherwise be so large that it would make reading the data difficult for a similar reason as previous.
    The caption now reads:
    **“Comparison of code-based and interactive processing with datacleanr of raw sap flux data**. Compared to often more tedious, code-based filtering, the interactive quality control using domain expertise allowed retaining more observations resulting in greater data coverage across days (x-axis) for the measurement campaign, which lasted a total of 2769 days. Here, three additional full days of measurements, as well as several additional days with varying partial coverage were retained, as indicated by text labels to the right of bars (completeness by day; e.g. 25 % of all measurements for a given day). This is because individual, problematic observations could be removed interactively (see inset), which may increase explanatory power in subsequent analyses.”

### SI:

I like the SI a lot and the gifs work brilliantly - thanks.

Thank you for recognizing and noting.

- SI: Example 2 has missing GIF
  - The SI is somewhat slimmed down from the GitHub ReadMe due to the file size limitations of PLOS. We opted to provide the code for such examples with missing GIFs regardless, as it presents a helpful starting point. We added notes such as “similar execution as above” or, where available, a link to another existing animation. We hope this satisfies the concern, while also making the SI more user-oriented.

### General comments (which do not require any action from your side)

- text based data object versus RDS: most data is stored in a csv file, as it can be generated from e.g. Excel. So using these as input would be very useful. Export of results does not need to be lossless, ut only include relevant results from the result and additional info could be saved as text files with additional details or even rds files. Saving should be lossless, so RDS is here appropriate. CRAN issues - use RUniverse for "full" package, provide functions in the package to download the additional info and data when needed.
  - Thank you for highlighting this – food for thought and perhaps a starting point for a revamp in a future version!
- l 269: If you put the shiny app on shiny server, you can use it from all platforms (even smart phones) which have a browser. Probably include this in future plans (not relevant to the paper here).
  - This is a good point, and could be interesting to actually call the remote shiny app with dcr_app(), rather than sarting a local server. That would likely also overcome some issues with XQuartz for rendering static visualizations in the summary. I will look into this also – thank you for the suggestion.
- Reading in from databases (sqlite and duckdb come to mind as widely used stand alone databases which are used for larger datasets when standard handling in R is not possible anymore) would be extremely useful as a next step.
  - Again great suggestion, and thanks for highlighting. I will start learning SQL in the near future, so I am motivated to look into this also.
